# Targeted Nanotheranostics: Integration of Preclinical MRI and CT in the Molecular Imaging and Therapy of Advanced Diseases

**DOI:** 10.7150/ntno.95791

**Published:** 2024-04-23

**Authors:** Jyoti Bonlawar, Aseem Setia, Ranadheer Reddy Challa, Bhaskar Vallamkonda, Abhishesh Kumar Mehata, Vaishali, Matte Kasi Viswanadh, Madaswamy S Muthu

**Affiliations:** 1 Department of Pharmaceutical Engineering and Technology, Indian Institute of Technology (BHU), Varanasi-221005, India.; 2 Department of Pharmaceutical Science, School of Applied Sciences and Humanities, VIGNAN'S Foundation for Science, Technology & Research, Vadlamudi, Andhra Pradesh, India.; 3 Department of Pharmaceutics, KL College of Pharmacy, Koneru Lakshmaiah Education Foundation, Greenfields, Vaddeswaram 522302, AP, India.

**Keywords:** magnetic resonance imaging, computed tomography, nanotheranostics, advanced diseases

## Abstract

The integration of preclinical magnetic resonance imaging (MRI) and computed tomography (CT) methods has significantly enhanced the area of therapy and imaging of targeted nanomedicine. Nanotheranostics, which make use of nanoparticles, are a significant advancement in MRI and CT imaging. In addition to giving high-resolution anatomical features and functional information simultaneously, these multifunctional agents improve contrast when used. In addition to enabling early disease detection, precise localization, and personalised therapy monitoring, they also enable early disease detection. Fusion of MRI and CT enables precise in vivo tracking of drug-loaded nanoparticles. MRI, which provides real-time monitoring of nanoparticle distribution, accumulation, and release at the cellular and tissue levels, can be used to assess the efficacy of drug delivery systems. The precise localization of nanoparticles within the body is achievable through the use of CT imaging. This technique enhances the capabilities of MRI by providing high-resolution anatomical information. CT also allows for quantitative measurements of nanoparticle concentration, which is essential for evaluating the pharmacokinetics and biodistribution of nanomedicine. In this article, we emphasize the integration of preclinical MRI and CT into molecular imaging and therapy for advanced diseases.

## Introduction

In the recent era, preclinical research on magnetic resonance imaging (MRI) and computed tomography (CT) based nanotheranostics has gained significant popularity for diagnosing various diseases such as cancer, cardiovascular diseases, brain tumors, coronary artery disease (CAD), etc 1. All imaging modalities, in particularly CT, which is employed for morphological visualization, positron emission tomography (PET), which has exquisite specificity for molecular imaging of metabolism and MRI which is used for morphological, physiological, and functional data in humans, can all be used similarly for small animal imaging 2, 3. Small animals are important in preclinical research and they are helpful as models of human disease 4. Preclinical is the term used for an evaluation done on small animals before the clinical trials in humans 5. Preclinical imaging such as MRI and CT scans helps us to understand animal models as well as plays pivotal role in treatment 6. Different types of animals such as mice, rats, rabbits guinea pigs, etc. are used for preclinical research 7. MRI is mostly used non-invasive and radiation free imaging modality which gives insight into human physiology 8. It gives a very good visualization of soft tissues; in-depth observation and evaluation of hard tissues can also be achieved with the help of contrast and embedding medium 9. In MRI, the behavior of nuclei under an external magnetic field generates net magnetization, resulting in detailed anatomical images 10. There are different types of magnetic resonance systems based on magnetic field strength such as high field 3 Tesla (3T), mid field 1.5T, and ultra-high field magnetic resonance having a strength of 7T. Currently 3T scanners are used as imaging modality in central nervous system (CNS) disease in humans and it was reported that as the strengths of magnetic resonance system increases their clinical application 11, 12.

The fundamental principle of CT is that X-rays are absorbed differently by tissues with varying electron densities as these ray's transverse through the body 2, 13. A slice of tissue is exposed to radiation via CT and the output is measured at a different location. The incident beam will attenuate to varying amounts to tissues having different densities. The slice is measured multiple times and a fast fourier transformation map is produced from different densities of tissue examined within the slice. The resulting data is transformed into an image for radiological analysis 14, 15. Clinical CT's fundamental components are a patient-supporting table, detectors, a moving X-ray tube, a computer, and a viewing console 16. There are various types of CT systems, including conventional or incremental CT, helical or spiral CT, and multi-detector or multi-slice CT 17. CT is a non-invasive imaging technique employed for evaluating the structure and functionality of the heart. 18. There are numerous recognized and novel uses for CT, including the analysis of ventricular morphology, natural and artificial valves, pulmonary and coronary vein anatomization, left and right ventricle function 19.

Multi-detector computed tomography (multi-detector CT) serves as a valuable alternative, particularly for patients who cannot undergo MRI when examining masses in the cardiac and para-cardiac regions 20, 21. High spatial and temporal resolutions of multi-detector CT make it sensitive to the presence of fat and calcification 22. It can identify which neoplasms are aggressive and benign, measure the size of tumors, and assess how masses impair heart function 20. MRI can be helpful in the detection of cardiac tumours 23, 24. MRI has the advantages of soft-tissue contrast and it provides high-resolution anatomical images as compared to CT 25. MRI is crucial in the diagnosis of obstructive coronary artery disease (CAD) 26. The absence of ionizing radiation and non-invasive nature makes it advantageous for evaluating heart anatomy, blood flow, tissue characteristics, and function, and diagnosing atrial shunts, ensuring patient safety. It is beneficial in assessing heart anatomy, blood flow, tissue features, function, and atrial shunt diagnosis because it is non-invasive and does not pose any risk of ionizing radiation to patients 19. Repeated longitudinal inspections and follow-ups of atherosclerotic plaques using MRI can also be helpful 27. Precise measurement of plaque size and thickness is made achievable by the exceptional contrast in soft tissues and the high-resolution capability of this method, which effectively distinguishes between vessel lumen and vessel wall 28, 29. After the bolus injection of gadolinium the first-pass MRI images were obtained, including gadolinium-diethylene tri-amine-penta acetic acid, with 91 percent sensitivity and 81 percent specificity, can be used to identify coronary artery stenosis 30, 31. High spatial resolution MRI was effectively used by Beuf *et al*. to examine the various anatomy of mammalian dental systems in vivo 11. Clinical cervical cancer staging also employs MRI and CT. The high resolution of soft tissues in MRI allows for easy visualization of the size of cervical tumors and lesions, making it a great tool for diagnosing cervical cancer in its early stages. For the diagnosis of late-stage cervical cancer, CT is more suitable due to its speed and clarity of images 32.

Nanotheranostic means nano-therapy along with diagnosis on the same platform. In addition to visualizing drug delivery and concomitant therapy, it possesses the capacity to facilitate precise delivery of medications to specific targets 33, 34. Hence it decreases systemic toxicity and improves the therapeutic index. The application of nanotheranostics to drug delivery has several benefits, including in vivo imaging, combinatory therapy, systemic controlled release, synergistic multimodality therapies, stimuli-responsive release, etc. 35, 36. When it comes to the translational examination of the safety and therapeutic characteristics of nanomedicines, animal models implemented in living creatures play an extremely important function 37. Theranostics in small animals completes the process of developing these theranostic nanosystems for clinical application 38. The results of these investigations are essential for predicting the likelihood that human studies will be successful. Among the different models available for the preclinical evaluation of medications, the laboratory mouse model is frequently regarded as the most reliable. Mice and humans have nearly identical genes and have similar disease traits 39. Different mouse models offer the selectivity required to accomplish particular investigational goals. Mice have benefits over other models because of their tiny size, cheap maintenance requirements, quick gestation period, and simplicity of genetic modification. The results of preclinical animal trials have been examined to gauge the effectiveness of various theranostic systems. Moreover, animal experimentation has demonstrated that the incorporation of nanoparticles within a theranostic framework leads to enhanced diagnostic and therapeutic capabilities 40. The majority of animal investigations have noted considerable improvements in the efficiency of the therapeutic or imaging modalities used in theranostic systems 41.

This review primarily emphasizes the latest advancements and the application of MRI and CT in preclinical studies, particularly in the area of nanotheranostics. This review centers on different kinds of preclinical MRI contrast agents including tissue/organ-specific agents, blood pool agents, extravascular fluid agents, and microcomputed tomography contrast agents such as iodine contrast agents, metallic contrast agents, lanthanide-based contrast agents, gold nanoparticulate based contrast agent employed in preclinical imaging of different types of diseases. We have also discussed nanoparticulate based dual imaging by preclinical magnetic resonance imaging (PMRI)/micro-computed tomography (MCT) and recent advances in PMRI/MCT preclinical imaging. This review also discusses the therapeutic applications of various nanotheranostic materials and their successful integration with diagnostics for the treatment of cancer and other life-threatening diseases.

## 2. Development of CT/MRI

The foundation for the development of MRI was laid by Edward Mills Purcell and Felix Bloch's research on nuclear magnetic resonance at Stanford University in the 1940s 42. Paul Lauterbur put forth the fundamental ideas of MRI in 1973, and Richard Ernst created the requisite image reconstruction algorithms in the middle of the 1970s. While Ernst was presented with the Nobel Prize in Chemistry in 1990 for his substantial contributions 43.

In the early 1970s, the integration of X-ray diagnostic and computer technology allowed researchers to generate in vivo 3D images of the human body for the first time. This field is known as CT 44. Hounsfield's team invented the original CT scanner, which entailed a time-consuming process of days to transform the raw data needed for a single scan or "slice" of the body into a reconstructed image. The rapid capabilities of modern multi-slice CT systems enable them to gather up to 4 slices of data in under 350 milliseconds and convert millions of data points into a 512 × 512 matrix image in less than a second 45. The fastest multi-slice CT scanners are capable of scanning the complete chest (40 × 8 mm slices) in 5 to 10 seconds. Over the past 25 years, CT has made great strides in terms of patient comfort, resolution, and productivity. With faster CT scans, more anatomy could be scanned in less time. The removal of patient motion artifacts like breathing or peristalsis is made simpler by faster scanning. Compared to before, CT scans are now quicker and more comfortable for the patient. A significant amount of research and development has been invested in optimizing image quality for confident diagnostics while minimizing X-ray dose 46. Traditionally, different gadolinium-based contrast agents, including gadobutrol, Gd-DOTA, and gadobenate, were utilized in clinical settings for MRI imaging 47. However, nano-particulate based contrast agents (extravascular, blood pool, organ-specific, metal-based, polymer, and lipid-based) are being developed for MRI and CT imaging. They provide the number of benefits over traditional contrast agents such as targeted imaging, the possibilities of cell tracking, and extended blood pool occupancy 48.

## 3. Conventional/generalized contrast agents for CT/MRI

Commonly used T1 contrast agents derived from gadolinium such as Gd-DOTA, gadobenate gadopentetate, gadavist, gadoxetate, gadoterate, Dotarem gadodiamide, gadoteridol gadoversetamide, gadopentetic acid dimeglumine , etc and iron oxide based T2 contrast agents such as Feridex I.V., Lumirem, Sinerem, Risovist are used for MRI 49. Gadolinium-containing contrast compounds can be structurally divided into two groups based on the kind of ligand. The first type of agent is a linear agent, which surrounds the ion with an extended organic molecule ligand and the second type of agent is a macrocyclic agent 50. Agents that are linear and macrocyclic can both be ionic or non-ionic 51. There have been nine intravenous Gd-CA drugs approved so far, eight in Europe and six in the US. They consist of one extracellular gadolinium chelate designed specifically for MR angiography (gadofosveset), two liver-specific contrast agents (gadoxetate and gadobenate), and non-specific extracellular gadolinium chelates (gadoteridol, gadodiamide, gadobutrol, gadoterate, and gadopentetate). During the initial minutes following bolus injection, liver-specific Gd-CA combines the advantages of hepatocyte selectivity and biliary excretion with tested extracellular contrast agent properties. A recently developed blood pool contrast agent called gadofosveset has been designed for vascular imaging 52. It is a molecule based on gadolinium that forms reversible bonds with albumin present in the bloodstream. This contrast agent offers a greater signal intensity and an intravascular image enhancement that lasts for at least an hour, unlike other extracellular agents 53. Blood pool agents like existing extracellular agents enable both dynamic imaging and extended steady-state monitoring of the vasculature. People with normal renal function experience a notably extended T_1/2_ 18 h compared to the pure extracellular imaging agent. The potential benefits of administering a smaller dose may be nullified by the much-extended duration of bodily exposure 51.

Kramer *et al.* identified the effectiveness of an administering only one dosage of gadobutrol (0.1 mmol/kg of BW) compared to a significantly more dose of gadoterate meglumine (0.15 mmol/kg of BW). The study revealed that gadobutrol performed as effectively as a 1.5-fold of gadoterate meglumine in enhancing signals and delineating intra-axial brain tumors 54.

Contrast agents containing barium and iodine are currently being utilised for in vivo CT imaging in standard clinical practice. Due to their intrinsic high toxicity, barium-based contrast media are only permitted for use in oral imaging of the gastrointestinal tract. In the realm of X-ray CT imaging, iodinated contrast medium (ICM) has emerged as the predominant intravenous medium of choice 55. Different iodinated contrast agents such as iodixanol (Visipaque), ioxaglate meglumine (Hexabrix), iopromide (Ultravist), Iohexol (Omnipaque), iothalamate (CystoConray II) and iopamidol (Isovue) are used conventionally for the CT imaging 56. Small molecular ICMs continue to be the primary contrast agents used in clinics, but they still have rapid renal clearance and significant side effects, including acute renal toxicity, poor targetability, and low sensitivity. Organic nanoparticles, such as polymeric micelles, micelles, polymeric nanoparticles, nanoemulsions, polymer conjugates, dendrimers, and liposomes have emerged as highly favorable vehicles for delivering ICM due to their excellent biocompatibility, versatility in preparation and modification, and uncomplicated drug loading. In this way, nanomaterials are important for contrast agents that consists of tissue targeting properties, extended blood circulation time, lower renal toxicity, improved delivery, multimodal properties, improved sensitivity, and a time-flexible platform for next-generation contrast agents 57-59. As a testament to the outstanding therapeutic capabilities of iodinated nanotheranostics Fu *et al.* reported the facile synthesis of iodinated polyaniline nanoparticles, for dual-modal CT and PA imaging-guided PTT. The nanoparticles exhibited excellent CT/PA dual-mode imaging capability in addition to appealing PTT efficiency in vitro on 4T 1 cell lines. Using NIR laser irradiation, the efficacy of in vivo photothermal treatment (PTT) was assessed in Balb/c nude mice with 4T1 tumours. After 10 minutes of 808 nm irradiation, the temperature at the tumour site rose 51.6°C in the nanoparticle group. On the other hand, the control group that received PBS had a much smaller temperature rise, reaching 32.1°C 60.

## 4. Nanoparticle based MCT/PMRI of small animals

The area of surface to volume ratio of nano-imaging agents is much larger as compared to conventional contrast agents, allowing for surface labelling using specific ligands and molecules to improve both imaging capabilities, their pharmacokinetic profile and therapeutic outcomes 61. Nanoparticles have been proven to improve retention effects and permeability in solid tumours. Moreover, contrast agents display potent efficacy because of their extended circulation time (due to lymphatic drainage) and small size. Compared to single molecule-based contrast agents, nano-particulate probes demonstrated remarkably better medical imaging and detection capabilities 62. Functional visualization and the examination of biological processes are additional advantages of nanoparticle imaging 63. The interest in nanotechnology for biomedical imaging is rising quickly due to the drawbacks of traditional imaging contrast agents and the potential benefits of nanoparticle-based contrast agents for early diagnosis and microstructure visualization 61.

### 4.1 Preclinical Magnetic Resonance Imaging (PMRI)

PMRI is based on the fundamental principle of measuring the net magnetization induced by the mobility of water protons in a strong uniform magnetic field, which is regulated by gradient electromagnetic fields and radiofrequency pulses. Inductive coils can monitor this processing transversal magnetism and utilize it as the premise for reconstructing the image. The measured signal deteriorates over time based on two relaxation processes. The difference in MR images is caused by the fact that these relaxation durations depend on the tissue and that various organs produce distinct signals 64.

Small animal MRI studies, conducted in preclinical settings, often investigate or explore newly discovered disease processes that remain incomprehensible in humans. Most preclinical MRI machines run at field strengths between 4.7 and 11.7 Tesla. Although it is obvious that a higher signal-to-noise ratio is produced by a higher field strength, this also makes it possible to acquire data with a higher resolution 65. However, these experiments are significantly hampered by a number of artifacts and restrictions related to the particular absorption rate. Experimental animals have been subjected to various advanced MRI techniques, including functional MRI, perfusion, susceptibility weighted imaging, diffusion, chemical shift imaging, hetero nuclear imaging, and ^1^H or ^31^PMR spectroscopy in addition to conventional T1,T2, and T2*-weighted MRI techniques. Experimental studies exclusively utilize various MRI techniques, including those employing manganese-enhanced MRI and negative contrast materials, which are specifically designed for molecular/cell-specific imaging purposes 66. Various contrast agents for the MRI with their application have been presented in Table 1.

#### 4.1.1. MRI Contrast Agents

The inherent properties of MR active nuclei can be modified and signal contrast can be altered by employing substances like macromolecules, nanoparticles, supramolecular aggregates, small molecules etc. collectively known as MR contrast agents 80. Although, MR contrast agents do not actively participate in signal generation during MRI; rather, they influence the signal generated by other molecules predominantly those in close proximity to water molecules. In order to improve the distinction between healthy and infected tissues, MRI employs the contrast agents 80. Contrast-enhanced MRI has become increasingly crucial in modern radiology due to the expensive utilization of magnetic resonance imaging contrast agents (MRICAs) since 1988. Typically, the T1- or T2-weighted MRI modes have been employed to capture the remaining magnetization by adjusting the parameters in transverse or longitudinal planes, respectively. In contrast to the T2-weighted MR images, which reveal dark signal contrast, the T1-weighted MR scan displays recovered magnetization as brilliant signal contrast (residual magnetization) 81. The MRI contrast agents can be classified according to the following characteristics such as chemical make-up, magnetic property, presence of metal atoms, delivery method, impact on magnetic resonance imaging, bio-distribution, and applications.

In a study, Veiseh *et al.* demonstrate that a nanoprobe may penetrate the blood-brain barrier and target brain tumours selectively. A biocompatible polyethylene glycol-grafted chitosan copolymer coats an iron oxide nanoparticle, which is then coupled to a near-IR fluorophore, a tumor-targeting drug, and chlorotoxin to form the nanoprobe. The nanoprobe remains retained in tumours for an extended period and has a benign toxicity profile. This nanoparticle platform has immense promise for the detection and therapy of various tumour types, owing to its varied targeting ligand and flexible conjugation chemistry for alternative medicines 82.

Moreover, Henderson *et al.* identified that Au@SiO2@Au nanomatryoshkas (NM) are a very promising nanostructure. They demonstrate that by enclosing both kinds of contrast agents in the internal silica layer between the Au core and shell, a near-infrared-resonant NM may give simultaneous contrast enhancement for T1 MRI and fluorescence optical imaging (FOI). Additionally, they demonstrated that this T1 enhancement approach works even better with Fe(III), a contrast agent that may be safer than Gd(III) in some ways. The feasible option that would eliminate Gd(III) patient exposure: contrast agents based on Fe-NM have relaxivities that are 2 times higher than those of the frequently used gadolinium chelate, Gd(III) DOTA. Aside from a near-infrared photothermal therapeutic response, this dual-modality nanostructure can provide tissue visualisation with MRI and fluorescence-based nanoparticle tracking for assessing nanoparticle distributions in vivo 83.

#### 4.1.2 Extravascular fluid Agents (Intravenous contrast agents)

Extracellular fluid (ECF) agents are often known as intravenous contrast agents, which diffuse through the extracellular environment. These substances have been used for liver imaging for the longest time and still, they are the most renowned and extensively researched. Gadolinium chelated to an organic material, such as diethylene tri-amine-penta-acetic acid (DTPA) was used to make ECF agents 84. These contrast agents move around in the extracellular space before dispersing freely. Gadolinium is readily dispersed into the interstitium after entering the liver via the hepatic artery and portal vein. Apart from iodine molecules, which are detected using CT, gadolinium is detected using MRI rather than the molecule itself. The material has an amplification effect because a single gadolinium atom relaxes multiple neighboring water protons. As a result, MRI is more responsive to gadolinium implications than CT is to iodine effects. ECF agents are primarily excreted by the kidneys 85.

**Figure 1:**
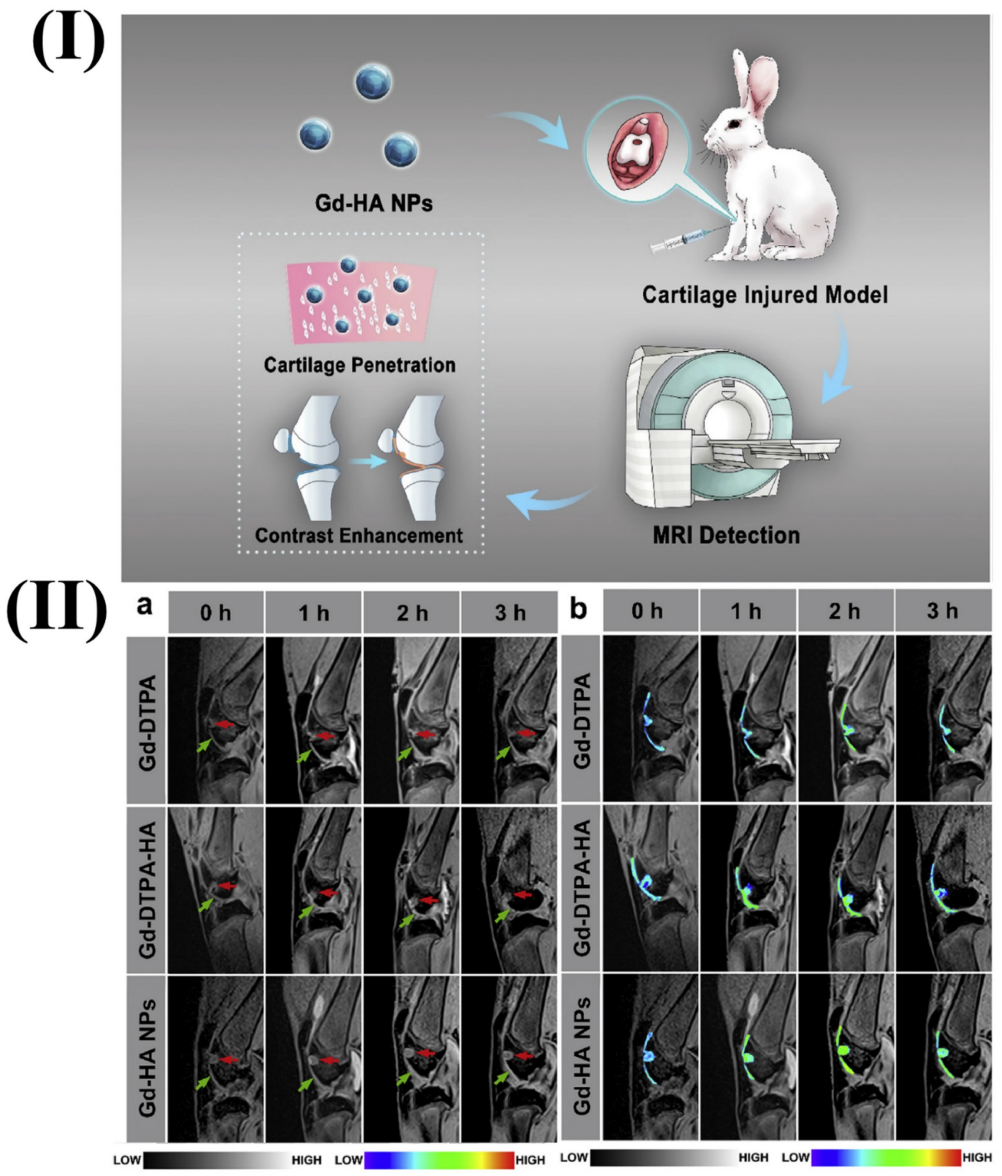
**I)** Illustration showing the use of Gd-HA NPs in MRI for the diagnosis of cartilage damage. **II)** In vivo MRI images of rabbit cartilage injuries after treatment with Gd-DTPA, Gd-DTPA-HA, and Gd-DTPA-NPs. Reproduced with permission from the ref. 86, Fig. 4 (Elsevier, 2020©).

In a study, Lu *et al.* fabricated gadolinium hyaluronic acid nanoparticles. In this study the rabbit model was used, its knees were injected with gadolinium-hyaluronic acid nanoparticles, gadolinium-DTPA-hyaluronic acid, and gadolinium. After the injection, the knee was imaged using a coil-equipped Siemens Verio 3T MRI system. The signal intensity changed at the cartilage injury site after treatment with Gd-HA NPs, Gd-DTPA, and Gd-DTPA-HA are shown in Fig. 1II. After an hour of Gd-DTPA injection, the articular cavity's signal intensity increased, and the lesion image slightly improved, but the cartilage's contours still remained unclear. At 2 and 3 h after injection, the proximity of signal intensities between the cartilage and the injured areas was maintained. However, in the case of Gd-HA-NP and Gd-DTPA-HA groups after 1h of injection, intense signals were observed. Additionally, the MRI signals for the lesion and cartilage were substantially greater after 2 h after injection. The intensities of MRI signals were seen to be gradually dropped after 3 h of injection indicting the metabolization of contrast agents. This finding was consistent with the established MRI time frame of 2-3 h for cartilage assessment. Gadolinium-hyaluronic acid NPs penetrated the core of cartilage tissues and functioned as an effective MR imaging probe for the precise diagnosis of cartilage injury because of their ideal size and great affinity for ECM (Extracellular Matrix). The research revealed that the nanoparticles could be useful for MRI-based clinical cartilage injury identification 86. Another study reported the fabrication of a new Gd-based nanoparticle for T1-weighted MR imaging called as dual-Gd liposomal agent. This agent combines the characteristics of core-encapsulated Gd liposomes (CGdL) and surface-modified Gd liposomes (SGdL) to deliver a large Gd concentration which increases signal intensity for each particle. The formulations SGdL, CGdL, and dual-Gd were given identical lipid doses of 200 mg/kg, resulting in equivalent blood levels of liposomes *in vivo*.

Similar contrast levels and brightness were applied during the processing of all maximum intensity projection images. The visualization of larger vessels was exhibited by all three agents but dual-Gd and SGdL liposomes displayed enhanced characteristics of smaller vessels. In the Dual-Gd photos, there was an increase in signal and a comparatively lower background. Additionally, tiny vessels became even more noticeable. This research also reported the enhancement of contrast-noise-ratio and signal-noise-ratio by the dual-Gd liposomes during in vivo imaging 87.

Baek *et al*. reported macrocyclic Gd-chelate, in which the ethoxy benzyl functional group was connected to 1,4,7,10-tetraazacyclododecane-1,4,7-trisacetic acid (DO3A) coordination chamber, exhibiting superior r1 relaxivity and chelation stability in comparison to linear-type Gd chelates. The *in-vivo* evaluation of liver-specific Gd chelate with a macrocyclic structure involved biodistribution analysis and liver MRI. The outcomes showed that the compound had a high tumour detection sensitivity which indicates that it could be a useful contrast agent for liver cancer imaging 88.

#### 4.1.3 Blood Pool Agents (Intravascular Contrast Agents)

Agents used in blood pools might be paramagnetic or superparamagnetic. The majority of paramagnetic materials are made of manganese and gadolinium, whereas iron oxide particles make up superparamagnetic materials 53. A blood pool agent's (BPA) characteristics that make it the best candidate for MR imaging include few immunologic side effects, little to no toxicity, initial distribution limitations to the intravascular area, a high T1/T2 relaxivity ratio, and uniform size 89. The BPA delivers robust & long-lasting vascular augmentation. BPA was developed to address the drawbacks of ECF contrast agents, as they have a prolonged retention period within the bloodstream and are removed from circulation much more slowly than their ECF counterparts. According to their mechanisms of action, the BPA has been categorized into three groups: (i) liposomes or polymers-based system that enhances the size of contrast agents, thereby reducing their leakage via endothelial openings; (ii) nanoplatform based systems that alter the route of elimination and (iii) the noncovalent association between albumin and low-molecular gadolinium to inhibit rapid leakage into the interstitium 89.

Ghaghada *et al.* investigated the application of liposomal gadolinium for the contrast-enhanced MRI of the placental-myometrial interface and placental boundaries in rats during pregnancy. The liposomal gadolinium here was used as BPA (liposomal-Gd). Non-contrast MRI was also conducted for comparative purposes. Conventional non-contrast imaging and clinically authorized Gd-chelate contrast agents were unable to detect placental margins, while liposomal-Gd enhanced MRI allowed for precise visualization of essential placental characteristics, such as placental-uterine junction and the boundary of the placenta. The pre-contrast image displays the descending aorta and portions of the intercostal blood vessels. The post-contrast Gd-DETA image reveals limited image contrast between the blood vessels and the adjacent tissue because Gd-DETA diffuses into the interstitial space. The 30-minute span of scans gathered revealed no change in image quality. On the other hand, the images obtained after liposomal-Gd administration revealed distinct vascular enhancement and minimal tissue augmentation. The post-liposomal-Gd images showed greater peri-spinal vasculature because of the strong target-to-background contrast. The post-Gd-DETA study showed a larger intravascular enhancement in contrast to the post-liposomal-Gd trial 90.

Moreover, Spuentrup *et al*. investigated the potential of visualizing clots in cortical veins and micro sinuses with contrast-augmented magnetic resonance angiography in an animal model of cerebral venous thrombosis utilizing gadofosveset and advanced spatial scanning. Blood clots were visualized using various sequences, compared between them on an individual basis and their contrast-to-noise ratio was assessed. The utilization of 3D Magnetic Resonance Angiography (MRA) enabled high-contrast imaging of small clots in the cortical blood arteries and cerebral sinus. This imaging technique, helped by the administration of gadofosveset, provided remarkable spatial resolution 91.

In another research work, Hou *et al.* fabricated an improved dual modal nanodiamond manganese contrast agent for T1 and T2-weighted MRI. After 6 weeks of tumor production, hepatic tumors bearing mice underwent MRI. Manganese conjugated to nanodiamonds increased the efficiency of transverse and longitudinal relaxivity compared to unconjugated MnCl_2_ and clinical contrast agents. In hepatic cancer-bearing mice, nano diamond-manganese complexes performed better than conventional clinical contrast agents although simultaneously lowering the level of harmful free Mn2+ ions in the blood serum. The nano diamond-based technique for enhancing dual-modal contrast agents offers an effective way to improve the efficiency of both T1 and T2 MRI employing a single kind of paramagnetic ion and single nanocomposites 92.

#### 4.1.4. Tissue/organ specific agent

The following three factors should be taken into account as much as possible while developing tailored and organ-specific contrast agents. i) better amplifying effect due to the fact that contrast agents for low and medium fields are not suitable for high and ultrahigh fields; ii) tracers that are specific to organ or disease must accumulate at the specific site to attain elevated local concentrations; and iii) improvement of tolerance: the compound must be entirely cleared from the body as well as chemically and physiologically inert in order to increase tolerance. The superparamagnetic iron oxide nanomaterial (SPIONs) exhibits hydrodynamic sizes between 1 and >100 nm. The accumulation of large hydrodynamic SPIONs in the body restricts repeated imaging investigations and poses a limitation on clinical management 93. There are various limitations associated with these large SPIONs, such as their inability to allow for effective renal clearance following intravenous administration, which differs from GBCA elimination pathways. They can also create a negative contrast for an extended period.

**Figure 2:**
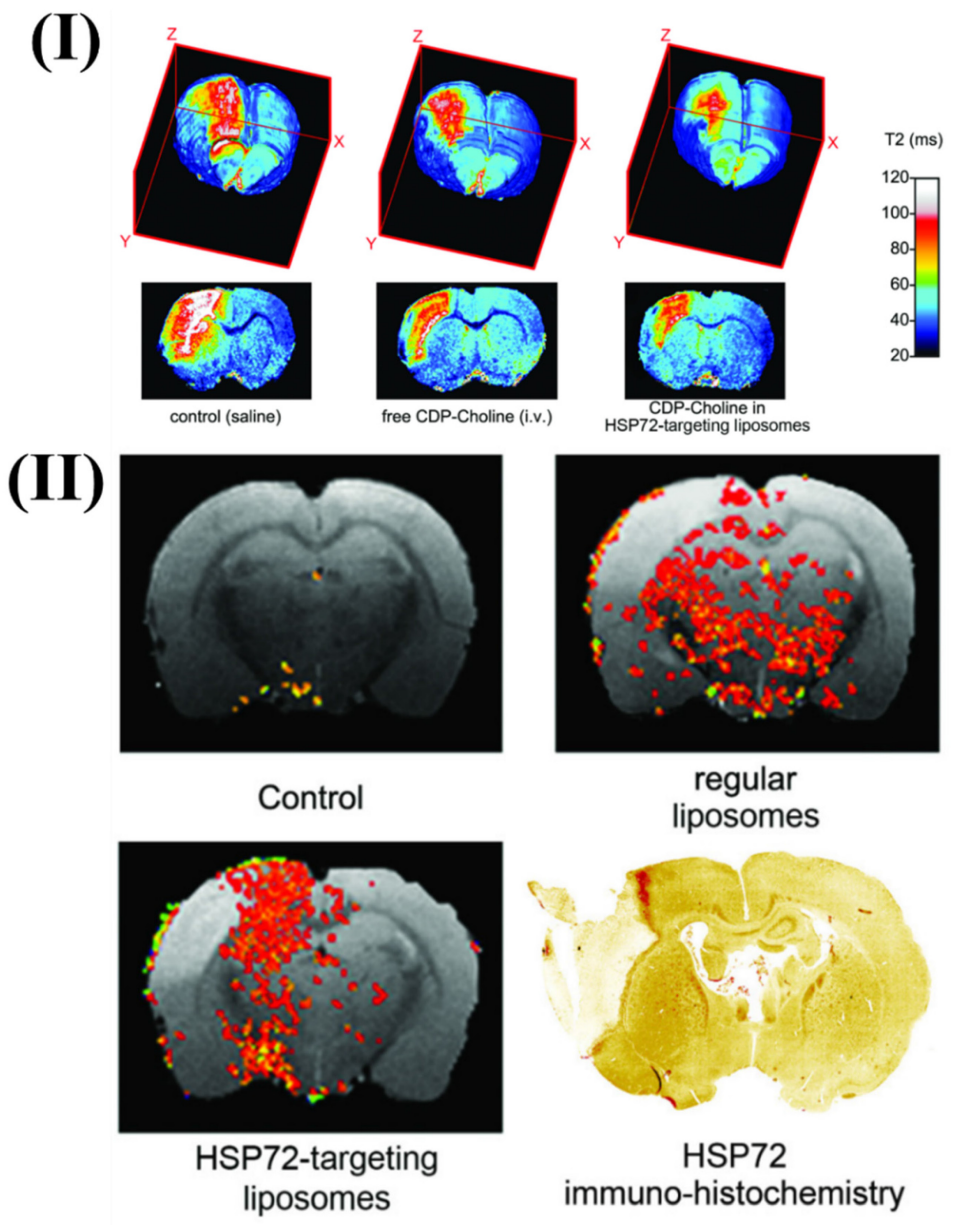
**I)** 3D color-coded magnetic resonance parametric images of three ischemic rats' brains showing transverse relaxation durations (T2). **II)** Comparative magnetic resonance imaging of rat brains after ischemia. Reproduced with permission from the ref 94 Fig. 6 and Fig. 8 (Theranostics, 2013©).

Agulla *et al.*, discuss the creation of a theranostic system for the purpose of treating stroke, which is the main cause of disability and mortality in industrialised nations. They also detail the process of creating anti-HSP72 vectorized stealth immunoliposomes that treated cerebral ischemia by enclosing a therapeutic substance (citicoline) and making them detectable by traditional imaging methods (fluorescence and MRI). By utilising MRI, they discovered that the majority of the vectorized liposomes (80%) were situated on the ischemic lesion's periphery. Interestingly, when animals were given liposomes, the lesion volumes were up to 30% less compared to animals given free (non-encapsulated) medicines. The promise of nanotechnology in the future of useful instruments for treating neurological illnesses is demonstrated by the results. More in vivo testing confirmed the theranostic agent's therapeutic potential. Enclosing citicoline in HSP72-targeted liposomes significantly enhanced the drug's therapeutic benefits, according to qualitative evaluations. Lesions on T2 parametric maps were smaller in animals given citicoline-loaded HSP72 targeting liposomes as compared to either the control group or animals given the free, non-encapsulated medication Fig. 2I. Moreover, Fig. 2II shows representative photos of the distribution of liposomes in the brain of rats that had an ischemia event 24 hours after the liposomes were injected systemically. The ischemic lesion's periphery is seen to be elevated in the anti-HSP72 vectorized liposomes, but animals treated with saline show no R1-enhanced areas (apart from a few scattered pixels that could be fitting noise). It is worth noting that ordinary liposomes, which are not targeted, seem to be dispersed randomly throughout both sides of the brain (Fig. 2II). An immunohistology picture of HSP72 expression in the brain of an ischemia subject is shown in Fig. 2II. The results demonstrate a robust relationship between the in vivo dispersion of liposomes and the ex vivo production of the protein, lending credence to the theranostic agent's molecular recognition capacity and diagnostic capabilities. While MRI scans show that HSP72 is highly expressed in a narrow band around the top of the ischemic lesion (Fig. 2II), a deeper inspection of the histology image reveals that the protein is overexpressed in a larger area surrounding the lesion. Because of the significant differences in sensitivity between the two methods, it may be challenging to establish a direct association between protein expression and liposome distribution in MR images 94.

#### 4.1.5. Polymeric based contrast agents

The visibility of particular tissues or organs during an MRI scan can be improved with the introduction of polymeric-based contrast agents. The polymeric structure of these substances contributes to the durability and effectiveness of the compounds 95. Dendrimers, polymersomes, or nanoparticles are only a few examples of materials that can be used to create the polymeric backbone of polymeric MRI contrast agents. The polymeric structure enables the attachment or encapsulation of paramagnetic or superparamagnetic ions or nanoparticles. Paramagnetic or superparamagnetic materials are included in the polymeric contrast agents. These elements produce a local magnetic field that improves contrast in MRI imaging because they have unpaired electrons within that create the field. Comparatively speaking, small molecule contrast agents are less stable and biocompatible than polymeric MRI contrast agents 96. The paramagnetic or superparamagnetic components are shielded by the polymeric structure, which stops their deterioration or release during blood circulation in the body. Additionally, these substances are often made to be biocompatible which lowers the possibility of adverse reactions. Polymeric contrast agents can be functionalized with certain ligands or antibodies to target particular tissues, cells, or receptors 97. This focused strategy increases the agent's concentration in the intended location, boosting the contrast and specificity of the MRI. Due to the prolonged circulation, a larger window of opportunity exists for taking high-quality MRIs. Depending on the desired use and the selected polymer, polymeric contrast agents can be engineered to have a variety of clearance routes, including renal clearance or hepatic clearance 98. In an effort to create more effective and specialized MRI contrast agents, researchers are actively investigating various polymeric structures, including dendrimers, micelles, and nanoparticles. These developments are meant to increase clinical imaging applications' sensitivity, selectivity, and safety. Different polymeric MRI contrast agents have been studied and used in preclinical imaging for their prospective applications. For instance, polymeric micelles have been thoroughly investigated as MRI contrast agents in preclinical imaging. In an aqueous environment, these nanoparticles self-assemble to form a core-shell structure. The hydrophilic shell offers stability and biocompatibility, whereas the hydrophobic core can include paramagnetic or superparamagnetic substances 99.

Highly branched polymers called dendrimers have well-defined sizes and topologies. Due to their capacity to incorporate paramagnetic or superparamagnetic ions or nanoparticles, they have been studied as MRI contrast agents. Targeting ligands can be used to functionalize dendrimers for certain imaging purposes 100. Iron oxide nanoparticles with polymer coatings are highly magnetic and have great MRI contrast-enhancing capabilities. These nanoparticles can be coated with biocompatible polymers to increase their stability, biocompatibility, and targeting abilities in preclinical imaging 101.

Liu *et al.* 102. formulated Fe_3_O_4_-based poly(lactic-coglycolic acid) nanoparticles with a surface-modified cyclic Arg-Gly-Asp (cRGD) peptide were synthesized for thrombosis detection. In a rat model of FeCl_3_-induced abdominal aortic thrombosis, the nanoparticles quickly and specifically aggregated under vascular endothelial cells on the thrombosis' surface in both *ex vivo* and *in vivo*. The nanoparticles bio-distribution in the in vivo experiment indicated that the liver and spleen's reticuloendothelial system's macrophages may absorb the nanoparticles. In the Fe_3_O_4_ -PLGA-cRGD nanoparticles group, the T2 signal at the mural thrombus started to decline 10 minutes after intravenous injection. The abdominal aorta's peripheral zones' signal obviously reduced and then progressively recovered till 50 minutes had passed. The abdominal aorta signal in the Fe_3_O_4_-PLGA-NPs group did not significantly change before and after injection 102.

Due to the inefficiency of currently available small-molecule MRI agents based on gadolinium, Luo *et al*. prepared two types of Gd-based macromolecular MRI contrast agents of N-(2-hydroxypropyl) meth acrylamide (HPMA) polymeric systems (the core-cross-linked pH PMADOTA-Gd and the linear one) (Gd). The goal of this work was to identify the ideal polymeric formulation for a powerful and biocompatible MRI contrast agent. The r1 values of both core-cross-linked and linear pH PMADOTA-Gd copolymers were 10.49 and 6.42 mM1s1 respectively, which was a 2-3-fold increase over DTPA-Gd. Animal investigations showed that two different types of macromolecular systems resulted in significantly larger tumor accumulation, far longer blood circulation times, and signal intensities in contrast to the linear and clinical ones. Better imaging findings were seen with core-cross-linked pH PMA-DOTA-Gd, which may have benefited from its greater MW than pH PMA-DOTA-Gd. Finally, toxicity tests conducted both in vivo and in vitro revealed that both macromolecular compounds were highly biocompatible 103.

Moreover, Gao *et al*. fabricated pH-sensitive polymeric micelles made up of PEG-poly (-amino ester)/(amido amine) entrapping iron oxide (Fe_3_O_4_) nanoplatform to function as pH-responsive MRI contrast agents for observing cerebral ischemia areas and acidic pathologic areas. 3.0 T MRI scanner a superconducting quantum interference device were used to examine the Fe_3_O_4_-loaded polymeric micelle. Fe_3_O_4_ nanoparticles were successfully encapsulated in the polymeric micelle at physiological pH 104.

#### 4.1.6. Lipid based contrast agents

There is a need for flexible methods to synthesize contrast agents that linked with targeting moiety, particularly in the emerging area of molecular and cellular MR imaging. The rise in the utilization of lipid-based colloidal structures, including micro-emulsions, liposomes, and micelles as vehicles for drug delivery 105. This is done with the aim of enhancing the drug's bioavailability and pharmacokinetic properties, achieving targeted delivery, facilitating the administration of hydrophobic drugs, creating multimodal contrast agents, and, notably improving the T1 and T2 lowering capability. Consequently, lipidic aggregates incorporating MRI contrast agents have emerged as an appealing choice. In an aqueous environment, lipids, which are amphiphilic molecules possessing both hydrophilic and hydrophobic properties, tend to spontaneously aggregate 106. The amphiphiles are organized in these aggregates such that the hydrophilic portions face the water while the hydrophobic portions group together. Lipidic particles can be modified by attaching targeted ligands and ensure the particles gather at the disease site. Fluorescence microscopy can easily include fluorescent markers. The morphology of the aggregate might range from micelles to micro-emulsions to liposomes 107, 108.

The colloidal particles have diverse applications in enhancing MRI, such as BPA for magnetic resonance angiography, where they exhibit extended circulation times. The application of this technique extends to identifying abnormal tissues characterized by increased vascular permeability because this condition arises in instances such as myocardial infarction, inflammation, blood-brain barrier breakdown, atherosclerosis, and malignant diseases 80. The lipidic colloids which are particle contrast agents can be used for drug delivery which can be modified with ligands to specifically target a molecular marker of 109. Lipidic nanoparticles also show significant promise for cell labeling. The use of lipidic nanoparticles as multimodal MR contrast agents is a relatively recent and exciting development 110.

The effectiveness of liposomal gadolinium diethylene tri-amine penta-acetic acid (DTPA) enclosed within vesicles of 70 and 400 nm was assessed by a team of researchers as a liver contrast agent for MRI in rats with hepatic metastases. The smaller 70 nm liposomal Gd-DTPA vesicles exhibited greater contrast enhancement, which aligns with the fact that they have a larger surface-area-to-volume ratio. Five blinded radiologists were able to significantly improve their ability to detect metastases using lipid-enhanced images (P < 0.005). Conversely, the use of unencapsulated Gd-DTPA hindered lesion detection and resulted in a statistically significant decrease in the contrast between tumor and liver (P < 0.01). Liposomal Gd-DTPA exhibited sustained enhancement in vascular function for up to one hour following administration. The study demonstrated the potential utility of paramagnetic liposomes as a contrast agent for MR imaging 111.

**Figure 3:**
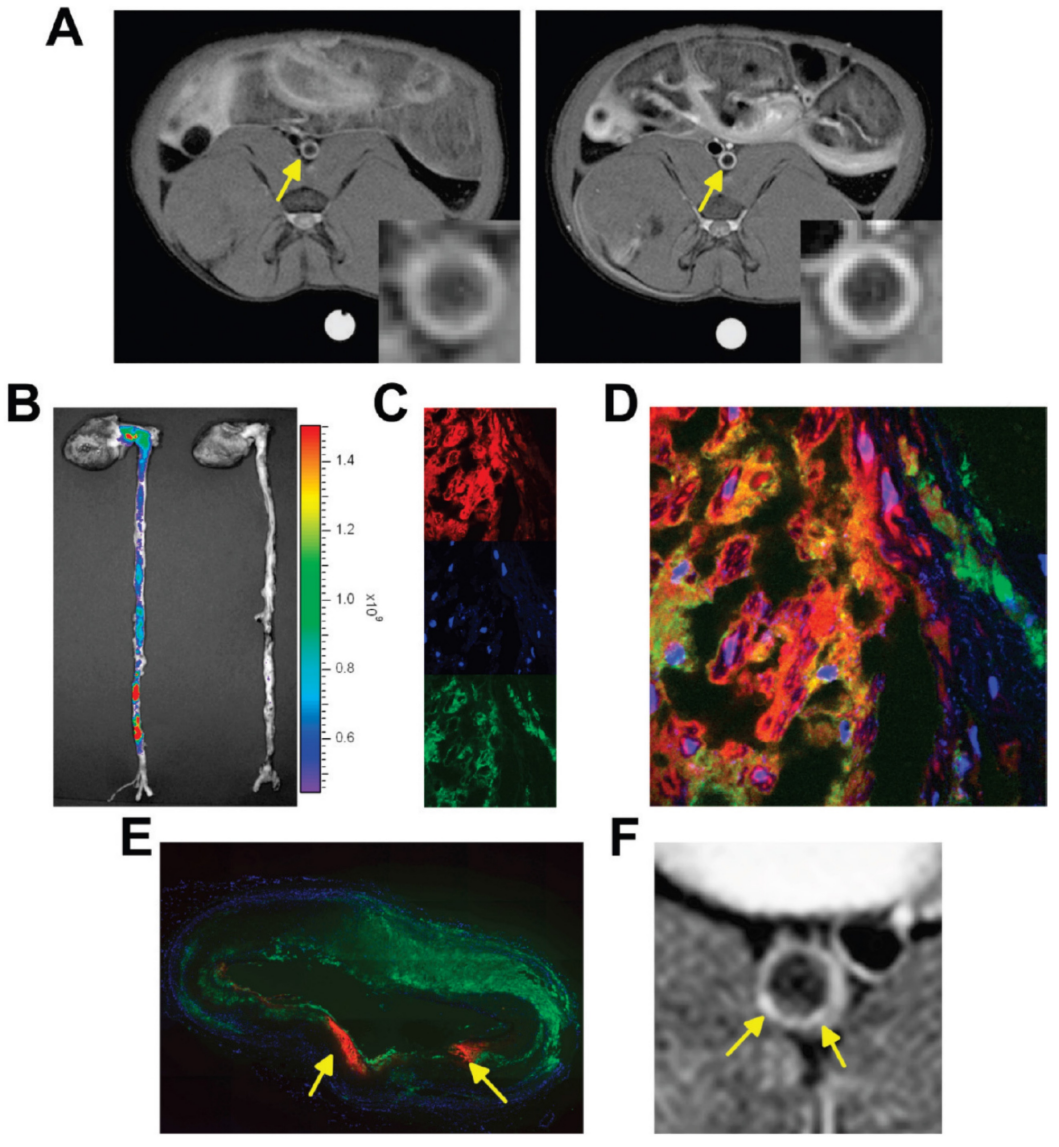
By using CLSM and MRI, images of the delivery and localization of liposomal PLP were obtained. Reproduced with permission from ref. 112 Fig. 2, (ACS Publications, 2010©).

Moreover, Lobatto *et al.* prepared a liposomal formulation for pre-clinical imaging. To improve the anti-inflammatory benefits of glucocorticoids (Prednisolone phosphate; PLP) while minimizing their side effects, a nanomedicine liposomal version of this drug (Li-PLP) was produced and administered *i.v.* (15 mg/kg) to atherosclerosis rabbit model. Oncology imaging techniques like dynamic contrast-enhanced MRI and ^18^F-Fluoro-deoxy-glucose PET were used to assess treatment outcomes over time. Substantial anti-inflammatory benefits were observed as early as 2 days after a single dose of Li-PLP, and these benefits continued for at least 7 days. Free PLP-treated animals showed no discernible differences. Macrophage density immunohistochemistry investigations confirmed these results in the vessel wall. The movement of gadolinium-labeled liposomal PLP after intravenous administration in atherosclerotic New Zealand White (NZW) rabbits has to be monitored using a T1-weighted MRI of the abdominal aorta. The MR images were depicted in Fig. 3A depicts the aorta wall of a rabbit both before (on the left) and two days after (on the right) the administration of PLP liposomes. An obvious rise in signal strength was recorded across the entirety of the inflamed artery wall, which is indicative of a significant accumulation of liposomes within the atherosclerotic lesions. After two days of administration, it was determined that the vessel wall saw a mean signal increase of 27%, with a standard deviation of 12.5%. They used CLSM on various aortic sections from various animals, NIRF imaging on intact aortas, and liposome assays to delve deeper into the uptake and location of liposomes within the inflamed artery wall. These techniques were undertaken to gain a better understanding of the process. When they examined the mean concentration of liposomes per gramme of aorta tissue, they found that it was 175 μg after 2 days after intravenous injection and 120 μg at 7 days after each administration. Liposomes that were labelled with Cy5.5 were found to be dispersed throughout the whole atherosclerotic aorta, as demonstrated by NIRF imaging (Fig. 3B). For CLSM, the liposomes were labelled with rhodamine, and they were able to be recognised as red fluorescent patches in the sections of the aorta (Fig. 3C, D). DAPI was used to label the nuclei of the cells. The identification of macrophages was made possible by fluorescently labelled RAM-11 antibodies (Fig. 3C, green), while the presence of macrophages was not possible. In the fused CLSM (Fig. 3D), it was discovered that the majority of the liposomes were found to be linked with macrophages. Even though liposomes were discovered across the full aortic plaque in every part. An illustration of a reconstruction of a full aorta section is presented in Fig. 3E. The MR image that corresponds to this has been displayed in Fig. 3F 112.

Another study investigated the utility of MnCl_2_ encapsulated in liposomes as a liver-specific contrast material for MRI. Toxicity testing revealed that the efficient dose for imaging studies was 7 to 11-fold smaller than the LD_50_ of pure MnCl_2_. In rats with liver tumors, liposome-encapsulated MnCl_2_ improved the relaxation rate of the liver by 2-3 times but had no impact on the relaxation rate of cancerous tissue. T1 weighted, 0.5 T MRI of an R3230 adenocarcinoma of the liver after the administration of pure MnCl_2_ (25 µmol/kg) showed a significant rise in the signal intensity of both healthy liver and cancer tissue. In contrast, the liver showed a considerable rise in signal intensity after the injection (40 µmol/kg) of liposome-encapsulated MnCl_2_, while the signal intensity of the tumor tissue showed only a little change 113.

### 4.2 Micro Computed Tomography (MCT)

Micro-CT sometimes referred to as micro-tomography or micro-CT, is a 3D imaging method that employs X-rays to examine within the object. It scans images like those from hospital CT scans but at a much smaller size and higher detail. Scanning of objects up to 200 mm in diameter and imaging of samples with pixel sizes as fine as 100 nm can be performed with the help of micro-CT. Preclinical studies use it for high-resolution imaging of small animals. The micro-CT technology offers a non-surgical examination for checking physiological alterations in small animals. Economic viability, quick scan times, high bone and lung sensitivity, and excellent spatial resolution are some of the possible benefits of micro-CT 114.

A development in computed tomography called micro-CT allows for extremely high-resolution imaging in three dimensions on a small scale. It requires no sample preparation or histological slicing which is necessary in others. The interior structure of a tissue can be seen with a micro-CT scanner without damaging the sample tissue 115. Compared to serial histological analysis, micro-CT is more affordable because of less sample preparation and the frequent need for post-processing. Micro-CT has the benefit of not interfering with concurrent structural or biomechanical investigations on both fresh and preserved tissue. This feature distinguishes it as a well-established technology capable of supplementing or even replacing histological investigation in a variety of scientific applications. High-resolution CT images of small animals and specimens are now achievable during scientific research owing to current technological breakthroughs, including the accessibility of megapixel charge-coupled device detectors and major improvements in computer capabilities and performance. In preclinical research, micro-CT has mostly been utilized to describe the phenotypic characteristics of knockout and transgenic animal models. However, now there are rapidly expanding applications in vascular studies with the quantification of density and architecture of bone that have been also explored 116.

Nanoparticle contrast agents are utilized in small animal micro-CT to prevent fast renal elimination. As for micro-CT imaging, a variety of nanoparticles have been employed, in most of the research has been concentrated on the utilization of gold and iodine-containing nanoparticles as contrast agents. Both types of nanoparticles can be targeted with a number of targeting mechanisms or operate as highly effective blood pool contrast agents 117. Contrast-enhanced micro-CT has various applications in the imaging of the heart, vasculature, malignancies, and abdomen in small animals. The focus of current research is on the development of micro-CT contrast agents with active targeting, multi-modal, or theranostic capabilities 118.

The ability of gadolinium nanoparticles coated with poly(ethylene glycol) (PEG) to function as a micro-CT vascular contrast agent was proven by Cruje *et al.* A concentration of 100 mg/mL of gadolinium could be achieved by lyophilizing the coated particles and re-dispersing them in an aqueous solution. Results from micro-CT scans taken 30 minutes after the contrast agent was injected intravenously into animals demonstrated blood pool contrast increases of 200 HU or higher. Quantitative study of gadolinium in tissues and imaging revealed that the contrast agent was present in the spleen and liver, two organs responsible for elimination, while other organs contained extremely minute levels. According to data collected from mouse body weight measurements, in vitro cell culture tests, and subcutaneous injections, the medicines showed minimal toxicity. The spleen revealed cytotoxicity in the histological examination of tissues conducted 5 days after the contrast agent injection; nevertheless, the liver, lungs, kidneys, and bladder appeared normal 119.

#### 4.2.1 Iodine Based CT agents

Iodine based contrast agents consist of water-soluble aromatic iodinated compounds as a building block. Due to their large atomic number and powerful photoelectric effect, these compounds offer great enhancement in contrast. Iodinated contrast agents are mainly classified into two types the “non-ionic” and “ionic” molecules. The majority of ionic contrast agents investigated so far are negatively charged species. These ionic contrast agents are commonly employed in the clinic. However, they have a number of limitations over non-ionic contrast media 120. The disadvantages of ionic contrast agents include interactions with biological elements like peptides and cell membranes, high inherent osmolality in their aqueous formulations, which could cause renal damage, as well as other physiological issues such as vasodilation, bradycardia, and pulmonary hypertension. Non-ionic iodinated imaging mediums are utilized to overcome the issues brought by excessive osmolality. These non-ionic contrast materials exhibit a decreased frequency of harmful health effects and a lower osmolality 121.

Currently, iodine remains a frequently utilized constituent for intravascular CT contrast in clinical settings. The utilization of clinical contrast agents in small animal imaging is particularly challenging because injected contrast agents are quickly eliminated in small animals as they have far higher renal clearance rates than humans do. BPCAs with extended blood residence times and steady augmentation for minutes to hours have been developed to overcome the typical contrast agents' fast elimination 122. One potential remedy to prolong blood retention, slow renal clearance, and stop "leakage" across capillaries is the use of Nano-sized contrast agents. Nano-sized iodine-based BPCAs consist of micelles, liposomes, iodine-containing polymers, and emulsions. These iodine nanoparticles have become the most often utilized contrast agents in micro-CT imaging. Commercially available iodine-containing BPCAs for preclinical research include ExiaTM (Binitio Biomedical, Inc., Ottawa, ON, Canada), Fenestra (Medi Lumine, Montreal, QC, Canada), Exitron TMP, and (Miltenyi Biotec, San Diego, CA, USA) 123.

In a study, Badea *et.al*. developed a liposomal-iodinated contrast agent for use with micro-CT to identify elementary lung cancers in mice. primary lung tumors were developed using KRAS mutations exclusively KRAS (LA1) or together with p53 (LSL-KRASg12d; p53fl/fl). In mice, a liposomal-iodine contrast medium comprising 120 mg Iodine/mL was administered systematically at a dose of 16 ml/g body weight. Longitudinal micro-CT scanning of cardio-respiratory gating was performed before and after dosing. Nodule sizes obtained from CT were utilized to evaluate tumor development. To investigate the dynamic enhancement of lung nodules, signal attenuation was assessed in individual nodules. The nanoparticle iodinated contrast agent allowed observation of the blood supply to the lesions during the early-phase scanning. Based on signal enhancement, delayed-phase imaging allowed for the characterization of nodules that were growing slowly and quickly. In comparison to KRAS(LA1) mice lung nodules, LSL-KRASg12d; P53fl/fl mice lung nodules showed significantly stronger signal enhancement. This study's longitudinal design also made it simple to image the prolonged tumor augmentation in LSL-KRASg12d; P53fl/fl mice. This study reported that the use of this agent may help with the early diagnosis and detection of pulmonary lesions and may also have an impact on treatment effectiveness and monitoring 124.

#### 4.2.2 Metallic Nanoparticles

 The nanoparticles which consist of the pure form of metals are called metallic nanoparticles. Most of the metallic based nanoparticles include iron, silver, gold, palladium, titanium, zinc, platinum, magnesium, alginate, and copper. Moreover, the most widely used theranostic nano-carriers are magnetic nanoparticles, which are made of an inorganic iron oxide, manganese oxide, or other magnetic substance 125. MRI or another imaging modality can detect magnetic nanoparticles inorganic core, which serves as a contrast agent. These inorganic nanocarriers multifunctional characteristics are achieved by surface-modifying magnetic nanoparticles with ligands targeting moieties or other substances 126. Clinical trials of several magnetic nanoparticles for use as MRI contrast agents to treat localised hyperthermia and as magnetically targetable medication delivery systems are now underway. Nanoparticles of iron oxide are one kind of such material 127.

##### 4.2.2.1. Iron oxide nanoparticulate contrast agents

Medical imaging, particularly MRI, makes substantial use of iron oxide nanoparticulate contrast agents, which capitalise on their unique properties. These agents improve the contrast of MRI scans of tissues by coats of biocompatible materials containing iron oxide nanoparticles. Their strong magnetic susceptibility amplifies signals, allowing for accurate tissue distinction 128. Targeted imaging applications like as tumour detection and biological process monitoring benefit from their small size, which allows efficient uptake by cells and tissues. The remarkable biocompatibility and low toxicity of iron oxide nanoparticles highlight their promise for use in diagnostic imaging in clinical settings 129.

**Figure 4:**
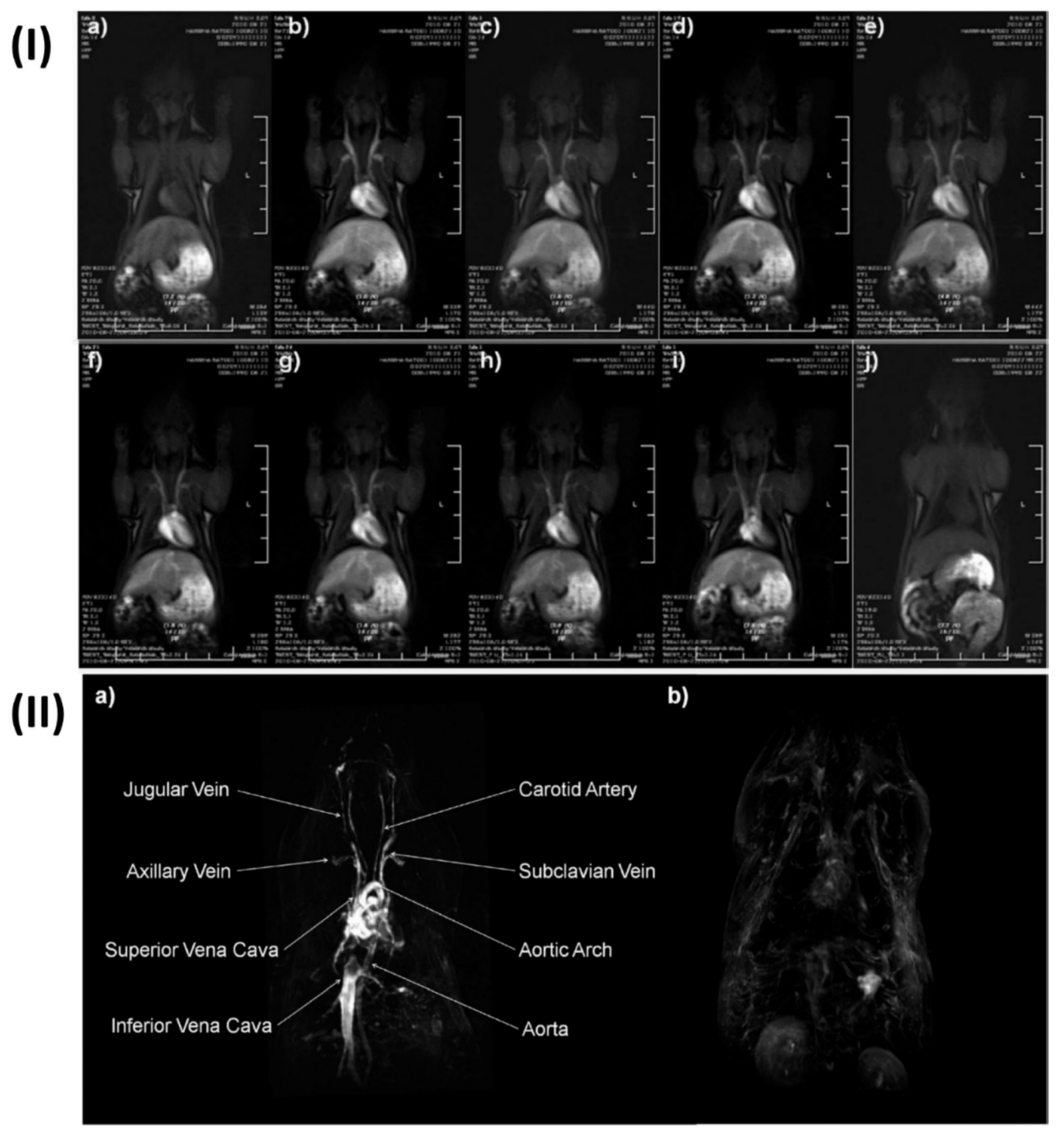
** I)** *In vivo* dynamic time-resolved MR images with ESFeON enhancement post-administration**. II)** (a) High-resolution MR pictures of a blood pool acquired using the 3d-FLASH sequence, augmented with ESFeON and (b) with DOTAREM images. Reproduced with permission from ref. 130 Fig. 4 and Fig. 5 (ACS Publication. 2011©).

Extremely small iron (Fe) oxide nanoparticles (ESFeONs) with diameters of about 4 nm were synthesized by Kim *et al*. used a heat-up approach to control the mechanical (temperature based) breakdown of iron oleate complex in the presence of oleyl alcohol. Maghemite crystal structure was discovered by the XRD pattern of ESFeONs. Due to their spin canting effect and modest magnetic moment, ESFeONs magnetization was significantly lower than iron oxide nanoparticles measuring 12 nm. Rats were given an intravenous injection of ESFeONs (2.5 mg Fe/kg) for in vivo MR imaging. T1 weighted images were captured at different time intervals. T1 weighted MR images displayed enhanced brightness of blood vessels after the injection of ESFeONs as shown in Fig. 4I, providing evidence of ESFeONs ability to enhance T1 relaxation in the circulatory system. The ESFeONs comprise low r_2_/r_1_ ratios of 6.2 and high r_1_ relaxivities of > 4.7 mM^-1^s^-1^ with a lot of surfaces Fe3+ ions containing five unpaired electrons were shown to be an effective T1 contrast agent. The application ESFeONs with high r1 relaxivity and extended blood circulation time allowed for the achievement of high-resolution T1-weighted MR imaging of various blood arteries with the capability to visualize the arteries as small as 0.2 mm diameters. Dynamic time-resolved MR angiography could retain the blood vessel's strong signal for 1 h Fig. 4I, demonstrated the viability of using ESFeONs as a T1-enhanced blood pool MRI contrast agent. The prepared ESFeONs' showed high r1 relaxivity, long blood half-life, low synthetic cost, and low toxicity. Hence in conclusion these nanoparticles were proven as efficient T1 MRI contrast agents for various clinical uses, such as the diagnosis of tumor cell angiogenesis, atherosclerotic plaque, renal failure, myocardial infarction, and thrombosis 130.

##### 4.2.2.2 Gold nano particulate contrast agent

Gold nanoparticle-based contrast agents exhibit 2.5-fold enhanced contrast at an equivalent concentration compared to conventional contrast agents made up of iodine or gadolinium used in medical applications 131. The contrast enhancement may be attributed to remarkable X-ray attenuation characteristics and a high atomic number of golds. Nowadays researchers are focused on modifying the gold nanoparticles and using them as a targeted drug delivery agent 132. Various structural alterations are coating nanoparticles with polymers, lipids, glycoproteins, etc., and creating them in core-shell structures. The modification of functional groups onto nanoparticles can change certain aspects of their performance, including the potential for dual-modal and/or multi-modal imaging applications, organ or tissue targeting ability, circulation kinetics, blood-pool residence, and stability 133.

Xiao *et.al*. described a simple method for preparing gold nanoparticles entrapped with acetylated dendrimers (GDENPs) that had more gold loaded inside the dendrimer. In contrast to a one-step complexation/reduction process, this method facilitated the synthesis of acetylated GDENPs with remarkable stability at dendrimer/Au salt molar ratios reaching up to 1:150. The formation of GDENPs was achieved through a sequential Au salt complexation/reduction method and subsequently acetylating the terminal amines of the dendrimer. Different approaches were used to characterize the newly generated GDENPs both before and after acetylation. According to TEM images, the synthesized GDENPs showed a fairly consistent size distribution with diameters that might vary based on the Au loading in the range of 2-4 nm. The produced acetylated GDENPs demonstrate remarkable stability under various pH and temperature conditions, with enhanced Au loading. The formulated acetylated GDENPs exhibited superior attenuation intensity in the computed tomography imaging when compared to Omnipaque, at the equivalent molar concentration of active elements (iodine or Au), and facilitated enhanced in vivo CT imaging of rat myocardium 134.

Another group of researchers synthesized gold nanoparticles that were embedded in PEG-modified branching poly-ethylene imine (PEI) for CT imaging of lung cancer, lymph nodes, and blood pool. In these gold nanoparticles, the initial molar ratio of PEI to Au salt was varied, and the residual surface amines of PEI were subsequently acetylated. The PEGylated PEI served as a useful template for entrapping uniformly sized gold nanoparticles with sizes ranging between 1.7 nm and 4.4 nm based on the molar ratio of PEI to Au salt. The PEGylated polyethylenimine-entrapped gold nanoparticles were selected for biological examination after optimizing the composition-dependent X-ray attenuation effect. The results demonstrated that the particles were cytocompatible within the specified concentration range and could serve as a contrast agent for efficient CT imaging of the lymph node of rabbits, lung cancer of nude mice, and blood pool in rats after intravenous administration. This research reported that produced gold PENPs with the right composition and dosage show tremendous promise for usage in CT imaging of various bio-systems 135.

#### 4.2.3 Contrast Agents Based on Lanthanide

Notwithstanding current developments in micro-CT, contrast agents based on iodine are primarily employed pre-clinically. Lanthanide-based contrast agents are challenging to manufacture at the elevated concentrations necessary for vascular imaging. However, higher atomic number elements like lanthanides, have X-ray attenuation qualities appropriate for spectral CT and can be used to achieve greater contrast 136. Lanthanide oxide (Ln_2_O_3_) nanoparticles are of particular interest in CT because they exhibit a high X-ray attenuation power and a considerable magnetic moment at room temperature. Moreover, surface-modified Ln_2_O_3_ nanoparticles display enhanced characteristics, including excellent colloidal stabilities, mild toxicities, and increased water proton spin relaxivities. The particle diameter of nanoparticles should be very small (3 nm) for in vivo applications to enable urine elimination from the body following intravenous injection. Such requirements are addressed by Ln_2_O_3_ nanoparticles, which exhibit high-quality MRI and CT imaging characteristics at extremely small particle sizes 137.

**Figure 5:**
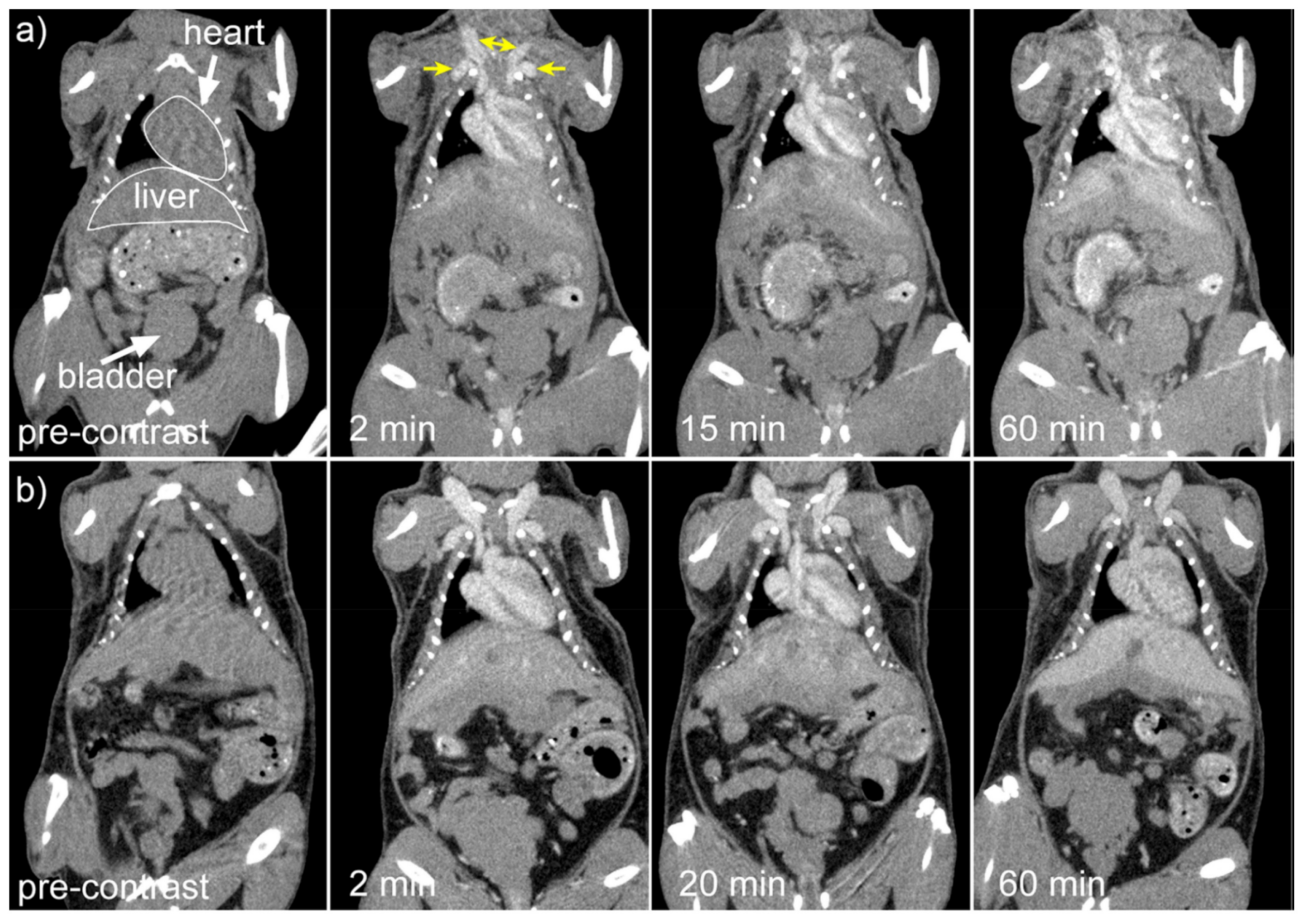
Coronal micro-CT pictures depicting the organs and structures of mice that were given a contrast agent formulated at a) 1:1 and b) 0.5:1 PEG114- PLA53:ErNP mass ratios. Reproduced with the permission from ref 136. Fig. 7 (ACS Publications,2018©).

Cruje *et al.*, created an erbium-based contrast agent that satisfies the criteria for micro-CT imaging, which include a stable colloidal structure upon redispersal of high concentrations, the capacity to avoid quick renal clearance, and circulation durations in tiny animals in the tens of minutes. The agent included 100 mg/mL of erbium. To fulfil the needs of live animal micro-CT, contrast enhancement of more than 250 HU was maintained in the blood pool for a maximum of one hour. Mice were scanned using three-dimensional micro-CT before and three times after each contrast agent formulation was administered intravenously. Obtaining each scan took sixteen seconds. In Fig. 5, shows micro-CT pictures that are representative of all the contrast agents. The external jugular and axillary veins, as seen in Fig. 5, were the most prominent after the contrast agent was administered. It seems like the contrast in the heart chambers didn't vary for both formulations for up to 60 minutes. The capacity to differentiate liver from surrounding soft tissue indicated the contrast in the liver as early as the 2-minute time point 136.

Moreover, Liu Y *et al.* fabricated a Yb-based contrast agent for CT imaging. PEG-UCNPs demonstrate negligible cytotoxicity and enhanced circulation time in vivo. Interestingly, under typical operating conditions, they showed more efficiency in comparison to traditionally employed Ta, Bi, Pt, and Au based nano-particulate contrast agents for CT and they also demonstrated significant efficacy in comparison to a clinically employed iodinated agent (120 kVp). The enhancement observed may be attributed to the higher energy position of Yb in the X-ray spectrum, especially K-edge energy. Additionally, these nanoparticles Gd doping gave them improved NMR and fluorescence imaging characteristics. These nanoparticles multimode imaging capacity improved the reliability of the data they collected and thereby can be employed for a variety of biological and medicinal applications 138.

## 5. Nanoparticle-Based Dual Imaging by PMRI and MCT

The development of multimodal imaging based nanotheranostics with enhanced accumulation within tumours is the key advancement in nanotechnology. Over the last decade, there has been great progress in the creation of multi-modal contrast agents based on NPs. Compared to administering various unimodal agents one by one, there are some practical benefits to using a single higher-order multimodal contrast agent 139. It is also helpful if the imaging contrast comes from only one source. Since multimodal agents have the challenge of demonstrating synergistic superiority over individual unimodal agents since optimisation and clinical implementation of unimodal drugs for different imaging modalities are already well underway. However, designing and synthesising NPs that can function in several modes is not an easy task. Integrating many modalities into a single agent increases the risk of functional or storage-related interference between the different contrast components 140. There are many benefits to both MR and CT imaging separately, however, it is necessary to produce a nanosized contrast agent with both MR and CT imaging components. Dual modal or multimodal imaging is required for precise or self-confirming disease imaging. Recently, only a few contrast agents have been produced for dual mode MR/CT imaging. A team of researchers reported the design, evaluation, and use of dendrimer-entrapped gold nanoplatforms loaded with gadolinium (Gd-DGNPs) for dual modal MCT-PMRI imaging. In this study, precursors used for the fabrication of gold nanoparticles were polyethylene glycol monomethyl ether and gadolinium modified five poly (amidoamine) dendrimers. Multifunctional Gd-DGNPs were produced by sequentially chelating Gd (III) and acetylating the remaining amine groups on the dendrimer terminals. The formed Gd-DGNPs exhibited 1.05 mM-1s-1 relaxivity for MR imaging by utilizing Gd (III) ions and X-ray attenuation characteristics (due to entrapped AuNPs) for CT imaging, that enabled simultaneous MR/CT imaging of some major organs (bladder, kidney, liver and heart) of rats and mice within a 45 min time period. Additionally, a study of biodistribution showed the Gd-DGNPs had a long blood circulation duration and could clear the major organs in 24 h. In their study, they showed that the adaptable dendrimer nanoplatforms facilitates the loading of both Gd (III) ions and AuNPs on the dendrimer surface. Furthermore, they concluded that the prepared Gd-DGNPs could serve as a theranostics nanoplatform for simultaneous MR/CT imaging with high sensitivity and high accuracy. Moreover, dendrimers possess distinctive structural traits, enabling further modification with different targeting ligands 141.

In a similar study, another group of researchers reported the fabrication of ultrasmall Fe_2_O_3_ nanoparticles incorporating macrophage laden gold nanoflowers (MLAuNFs) as a theranostic scaffold (Fe_3_O_4_/MLAuNFs) for enhanced simultaneous MR and CT imaging of cancer. The obtained Nano formulation had high cytocompatibility, and strong colloidal stability, performed well on CT and MRI imaging and the size was near about 90 showing reasonably good relaxivity of 1.22 mM**^-^**^1^ s**^-^**^1.^ Furthermore, MR/CT imaging efficiency of cancer utilizing Fe_3_O_4_/MLAuNFs was significantly improved compared to MLs after i.v. injection due to the effective transport of NFs to tumors with the support of MLs ability to avoid the immune system and target tumors. The finding suggested that the ML-mediated Fe_3_O_4_/MLAuNFs served as a multimodal contrast agent for cancer diagnostics 142.

**Figure 6:**
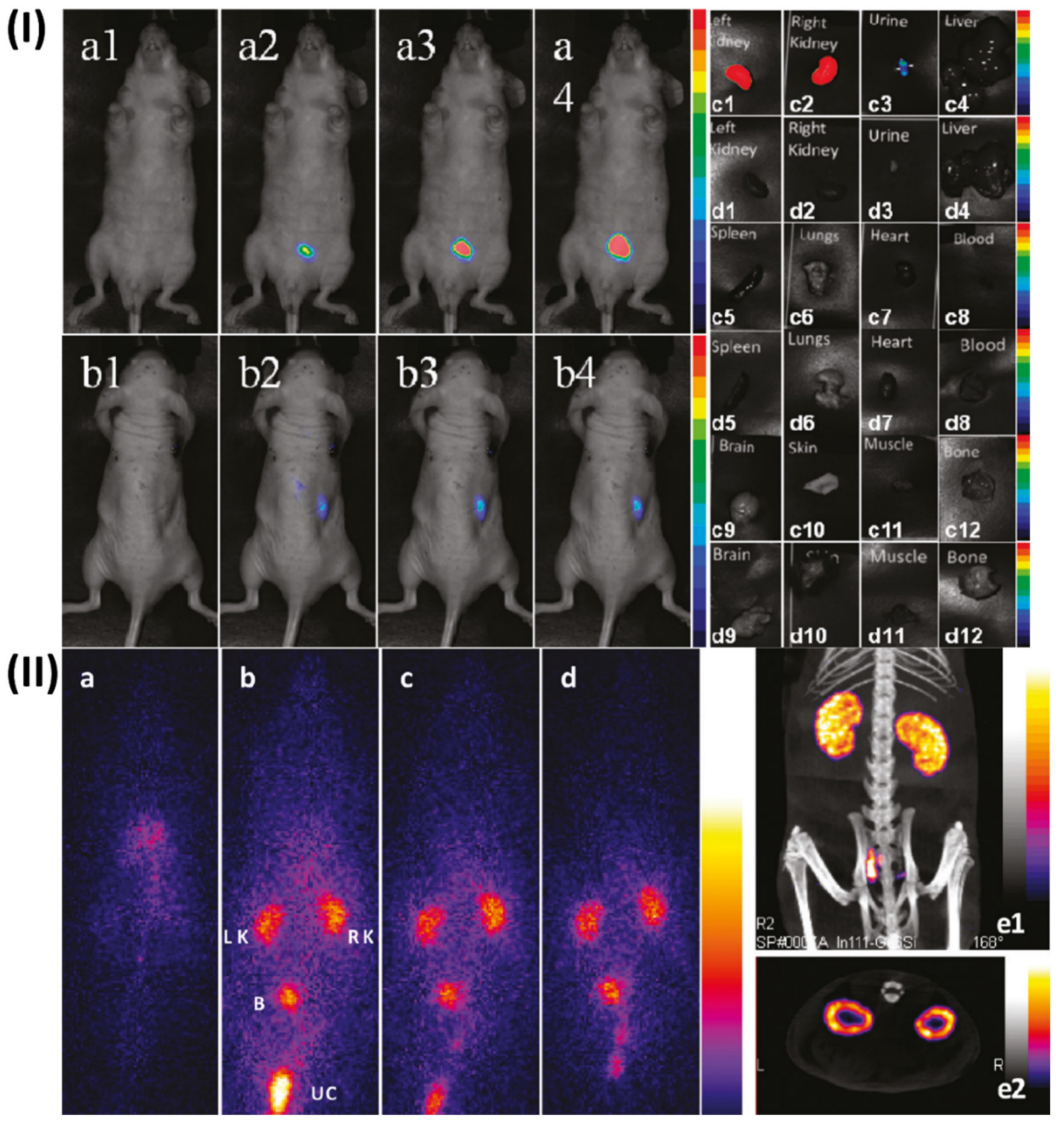
** I)** Dorsal and ventral fluorescence reflectance images after administration of Gado-6Si-NP. **II)** SPECT/CT in vivo imaging of wistar rat. Reproduced with permission from ref. 143 Fig. 5 and 7 (ACS Publications, 2011©).

Kryza *et al.*, determine the distribution of these multimodal Gado-6Si-NP in rats. Control Wistar rats and mice were scanned with SPECT, optical fluorescence imaging, and MRI (7T) after intravenous injection of Gado-6SiNP. When it came to renal elimination, SPECT, optical imaging, and MRI all showed a consistent association. The nanoparticles were found to circulate freely in the blood pool and were quickly eliminated by renal excretion, without building up in the liver or being taken up by RES, according to quantitative biodistribution that used gamma-counting of each organ that was sampled. These findings prove that Gado-6Si-NP have excellent biodistribution characteristics, which pave the way for their potential use as multimodal agents in in vivo imaging and theranostics, particularly in cancer research (Fig.6) 143.

Lipengolts *et al.* evaluated new core/shell Fe3O4@Au theranostic nanoparticles' capacity to enhance in vivo bimodal CT and MRI scans. Nanoparticles of core/shell Fe3O4@Au were produced and then coated with glucose and PEG. The nanoparticles were administered intravenously to C57Bl/6 mice that had Ca755 mammary adenocarcinoma tumours. MRI and CT scans were conducted on day 17 after injection, as well as at multiple intervals ranging from 5 to 102 minutes. Nanoparticles of core/shell Fe3O4@Au considerably improved tumour and tumour blood vascular function. Additionally, nanoparticles were collected and remained in the spleen and liver for 17 days. No harm was observed to the mice during the trial. Based on these findings, theranostic bimodal Fe3O4@Au nanoparticles are a safe and effective contrast agent for magnetic resonance imaging (MRI) and computed tomography (CT). Future biomedical uses of these nanoparticles may extend beyond cancer diagnosis 144.

## 6. Theranostic approaches for simultaneous imaging and therapy

Nanoparticles, loaded with some organic material alone or in combination with inorganic nano payload, have been developed which unveiled excellent pre-clinical outcomes with sensible features desired for photothermal/photodynamic therapy, thermal ablation, in addition to outstanding MRI/CT contrast (Table 2). Gadolinium based nanotheranostics showed enhanced cytotoxicity, apoptosis and inhibited tumor growth 145.

Xueyu *et al.* have developed gadolinium-apoferritin based nano delivery system which not only showed remarkable *T*_1_-weighted MRI performance and also produced excellent cytotoxicity in cancer cells *in vitro* and significant tumor growth inhibition via enhanced apoptosis and ferroptosis 154. Gadolinium porphyrin-loaded homogeneous supramolecular nanoparticles were effectively synthesised by Chen* et al.* Among the remarkable properties of these nanoparticles are their great longitudinal relaxivity, biocompatibility, long-term colloidal stability, dispersity, and fluorescence imaging ability. They also efficiently produce singlet oxygen (¹O₂). Moreover, these nanoparticles have demonstrated improved in vivo fluorescence (FL) and MRI capabilities, successfully directing the prevention of tumour growth in mice with CT26 tumours 155.

Murugan *et al.* have developed a new dual-therapeutic nanoparticle system with a core-shell structure that targets folate receptors. The barium titanate shell surrounds a molybdenum disulfide core. To make these nanoparticles more effective at targeting tumours, they were surface-functionalized with polydopamine (PDA) and then modified with folic acid (FA). In vitro tests using this functionalized nanoparticle technology showed that it was more effective at killing triple-negative MDA-MB-231 breast cancer cells 156.

Zhou *et al.* have synthesized iodine-rich semiconducting polymer nanoparticles for superior photodynamic therapy. As a photosensitizer and source of fluorescence signals, they made use of a semiconducting polymer that is sensitive to near-infrared light. Moreover, a nanocarrier and an iodine-grafted amphiphilic diblock copolymer (PEG-PHEMA-I) were used to boost the production of singlet oxygen (¹O₂). A 1.5-fold increase in the creation of ¹O₂ was the outcome of this improvement. The materials' high X-ray attenuation coefficient also made them useful in fluorescence imaging and dual-modal CT 157.

Chen *et al.* have fabricated MSN of tirapazamine with layer-by-layer formation of these supra-photosensitizers. The hyaluronidase (HAase) enzyme could induce the release of loaded therapies from this adaptable nanotheranostic platform, which could be preferentially taken up by tumour cells with an overexpressed CD44 receptor. Combinatorial PDT and bioreductive chemotherapy guided by NIR fluorescence/MR imaging resulted in substantial tumour suppression, with these nanoparticles accumulating preferentially at tumour sites in vivo 158.

To address tumour hypoxia with sensitised radiation therapy guided by precision imaging, Fan *et al.* created a theranostic nanoplatform. In this setup, dendrimer-entrapped gold nanoparticles complexed with gadolinium (III) were coupled with nitroimidazole, a hypoxia-targeting drug, using a polyethylene glycol linker (Gd-Au DENPs-Nit). Due to this layout, CT and MR imaging in two modes are now entirely feasible. The nanohybrids made of dendrimers and incorporating 3.2 nm Au cores showed good X-ray attenuation capabilities, a respectable r1 relaxivity of 1.32 mM⁻¹ s⁻¹, and improved uptake by cancer cells in hypoxia. This allowed for effective dual-mode CT/MR imaging of tumour hypoxia, which was a major outcome. In addition, when exposed to X-rays, these nanohybrids have the potential to produce reactive oxygen species, increase DNA damage, and hinder DNA repair. This could help with targeted radiation therapy for hypoxic cancer cells in the lab and hypoxia in tumours in the body 159.

Owing to ultrahigh X-ray attenuation coefficient, high NIR absorption coefficient, improved sensitivity in CT imaging, good biocompatibility and low toxicity, least expensive nature, etc., bismuth based nanosystems such as Bi_2_S_3_ nanorods, bismuth nanodots and semimetal bismuth nanoparticles shows to be competent photothermal cancer theranostic agents 131. To make the bismuth-based nanoparticles (Gd-PEG-Bi NPs), Wu *et al.* used pure bismuth nanoparticles and contrast agent gadolinium. The produced nanoparticles were very biocompatible and showed strong stability. Studies conducted in both laboratory settings and living organisms have shown that Gd-PEG-Bi NPs have an extremely high X-ray attenuation coefficient. This property causes MRI T1 relaxation durations to be short and photoacoustic imaging signals to be loud. After imaging diagnostics, Gd-PEG-Bi NPs successfully inhibited tumour growth in vivo when exposed to near-infrared laser radiation, thanks to their exceptional light-to-heat conversion efficiency 160.

**Figure 7:**
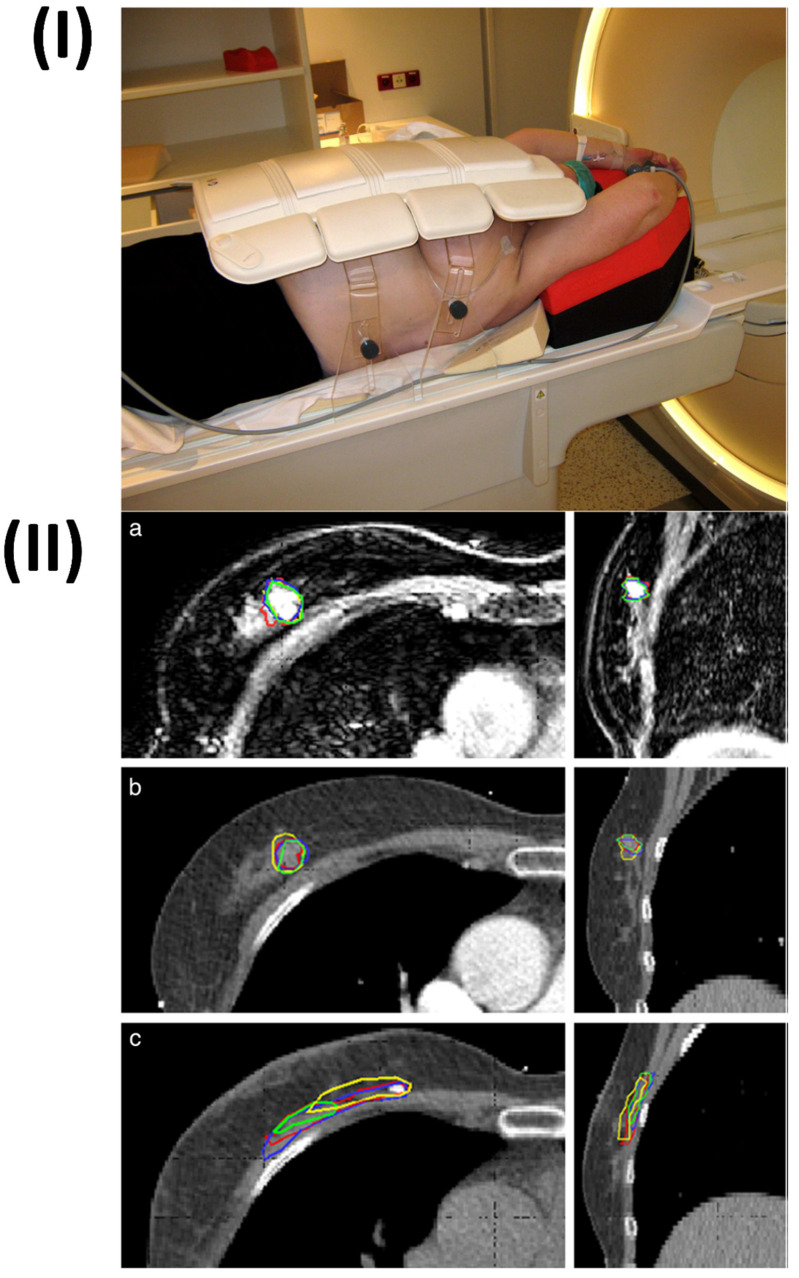
** (I)** MRI patient positioned in the supine position for radiation therapy; **(II)** 3D GTV outlines in the sagittal and transversal planes as seen by four separate observers on a single patient (a) Preoperative CE-MRI (b) Preoperative CE-CT (c) Clinical postoperative CT. Reproduced with the permission form ref. 161 Fig. 1, Fig. 3 (Springer, 2014©).

Hartogh *et al.* conducted the study on fourteen patients who were in the early stages of breast carcinoma. These patients had adenocarcinoma of the breast diagnosed as T1-T2, N0, and were scheduled for or awaiting sentinel node and lumpectomy treatments. In a recent development, four breast radiation oncologists were able to define tumours on preoperative contrast-enhanced (CE) MR and CT scans. A 1.5 cm margin surrounding the tumour was added to establish clinical target volumes (CTVs), excluding the skin and chest wall. The interobserver variability demonstrated conformity in the delineation of target volumes. Next, the volumes, dCOM (centre of mass distance), and conformance index (CI) were determined. An experienced breast radiologist scored tumour characteristics on CT and MRI scans. Both CT (0.80) and MRI (0.84) showed high median CI for the CTV due to preoperative tumour delineation. For the GTV, the CI was higher on MRI than on CT (p < 0.001), but for the CTV, it was not (CT (0.82) against MRI (0.84). Neither CT nor MRI showed any difference in the dCOM. The range of CT, CTV was 48 cm3 (p < 0.001), while MRI CTV was 59 cm3 (p < 0.001). The findings confirm once again that magnetic resonance imaging (MRI) is critical for the diagnosis of tumours as well as the visualization of abnormalities and spiculations (Fig. 7) 161.

## 7. Recent Advances in PMRI/MCT

Recent technological advancements in MRI equipment have resulted in the creation of sequences that incorporate metabolic and functional data in addition to anatomical information. In order to achieve preclinical imaging with a spatial resolution of even 20 to 50 μm, a combination of higher magnetic fields and high-sensitivity receiving coils, namely coils that are cryogenically cooled, has been utilized 162. The X-nuclei imaging and spectroscopy, quantitative imaging (relaxometry, diffusion tensor imaging,) time-resolved imaging (cardiac imaging with both retrospective and prospective gating), diffusion-weighted imaging, MR spectroscopy, and 7T MRI precise anatomical imaging are a few of the various advancements in preclinical MRI 163.

Functional magnetic resonance pulse sequences have recently been used to treat cancer, and they are now starting to improve the accuracy of clinical and preclinical diagnosis as well as tracking the efficacy of treatment of different types of tumors, including breast, prostate and brain 164. A new MR technique called 4D Flow combining 3D cardiac phase-resolved flowmetry with ECG-based phase-contrast MRI enables faster, more accurate, and more robust data acquisition. In this technique, morphological information is linked with functional information about a tissue's microenvironment 165, 166. The underlying tissue properties can be quantified using functional MR data 167. Functional MRI is a highly effective technique that offers comprehensive details regarding variations in the heterogeneity of treatment and lesion heterogeneity. It does this by combining objective biomarkers with morphologic data 168-170. In the initial stages of assessing treatment efficiency, functional MRI offers extra promise and should be helpful in drug development 171. Perfusion imaging and Diffusion-weighted MRI are two functional assessments that are already used in clinical settings. Other methods are still in the experimental stage, such as metabolic imaging with MRI 172-174.

Recently, X-ray detectors that are capable of counting individual photons or spectrum scanning with dual-energy micro-CT utilizing energy-integrating detectors have been developed in micro-CT. Photon counting X-ray detectors offer the capability to quantitatively differentiate intrinsic tissues by employing one or more exogenous contrast agents. The incorporation of it within a nanoplatform, such as liposomes, facilitates the emergence of innovative micro-CT imaging applications in the realm of cancer theranostics. X-ray phase contrast imaging is a significant field of micro-CT study. A wide range of new imaging applications can be achieved through X-ray phase contrast imaging because phase changes exhibit greater sensitivity to density fluctuations in soft tissues than traditional absorption imaging 175.

## 8. Future perspective

The ultrastructural examination of bodily tissues is now made possible by recent developments in magnetic resonance sequences and scanners, which also serve as key diagnostic and staging tools for tumor lesions. The utilization of magnetic resonance techniques in theranostics is leading to a novel application. This breakthrough arises from the capacity to take advantage of the magnetic characteristics of microparticles, previously utilized in the realm of nanotechnology 176. There are several questions that need to be addressed through in-depth basic research before most of these MRI contrast agents can be translated from preclinical studies into clinical applications 177. To address the most pressing questions, research teams from many fields must collaborate.

The future of imaging lies in multi-dimensional applications that add molecular and functional data to the anatomical imaging that CT is recognized for. In the future, spectral data will naturally be sampled in applications like coronary CT angiography employing PCDs. The measurement of calcium deposits in atherosclerosis investigations can then be improved by using this spectral information to eliminate image artifacts. Even non-invasive analysis of the content of atherosclerotic plaques may be possible by directing nanosized contrast agents to the plaque site. Increasing the robustness of these applications while limiting the radiation exposure to the patients will require more recent computing techniques, such as deep learning (DL) 175. Although there has been significant development, using organic nanoparticles to deliver ICMs is still a long way from being used in clinical practice. There are many issues that need to be tackled right away, including optimal pharmacokinetics, in vitro/in vivo stability, long-term biocompatibility, batch-to-batch reproducibility, and targeted distribution 178. Therefore, a lot of work is still required to produce effective organic nanoparticles for ICM delivery. Achieving a large-scale production of organic nanoparticles with precise and uniform shapes is currently a significant obstacle that needs to be overcome. Pharmaceutical and biomedical applications of nanomedicines and nanoimaging agents necessitate crucial factors such as consistent nanostructure, stable physicochemical properties, and reliable therapeutic performance. Crucially, the reproducibility of product quality and controllability of the preparation process play vital roles in meeting these requirements for successful biomedical and pharmaceutical applications 58. More efficient and accurate disease detection and treatment monitoring are the future of MRI and CT clinical trials. Better resolution, quicker scanning, and less radiation exposure are all on the horizon owing to technological advancements in imaging 179. There is hope for automated analysis, interpretation, and decision-making with the integration of AI algorithms with imaging data. Functional MRI and molecular imaging are two new methods that help researchers understand disease and its treatment better. Clinical trials that use MRI and CT will see revolutionary advancements in precision, speed, and data extraction in the future, which will allow for better, more tailored treatment for patients 180.

## 9. Conclusion

The fusion of preclinical MRI and CT techniques has enabled the advancement of tailored nanotheranostics. Combining the MRI and CT capabilities has allowed researchers to get valuable insight into the distribution, accumulation, and the therapeutic advantages and potential hazards of these nanoparticles. The pharmacokinetics and bio-distribution of nanoparticles have been thoroughly understood by researchers as a result of this combination, allowing for design optimization and improved therapeutic efficacy. Furthermore, the real-time monitoring and functional data provided by MRI, together with the high-resolution anatomical details and quantitative metrics provided by CT, have made it possible to precisely localize nanoparticles throughout the body and assess the effectiveness of therapy. These advancements have made it possible for personalized and precise therapeutic techniques in the field of nanomedicine, which have the potential to drastically alter how illnesses are treated and how patients are cared for. Preclinical MRI and CT integration has great promise for future targeted nanotheranostics research that will enhance drug delivery, expand imaging capabilities, and enhance patient outcomes.

## Figures and Tables

**Table 1:** Various contrast agents for in vivo imaging and their application.

Name of contrast agents	Classification	Molecular Structure	Target organ	Applications	Ref.
**Gadobutrol (Gadavist)**	Paramagnetic	Macrocyclic(Non-ionic)	CNS, Liver, Kidney, Breast.	Gadobutrol has been approved in India for MR angiography and MRI of the breast, liver, kidneys, and central nervous system (CNS) in patients 2 years of age and older.	67
**Gadoxetate disodium** **(primovist)**	Paramagnetic	Linear (Ionic)	Liver	Identification and classification of localized liver lesions in adults.	68
**Gadopentetate dimeglumine** **(Magnevist)**	Paramagnetic Extracellular	Linear (ionic)	Brain(CNS), blood vessels, and spin	It aids in the diagnosis of some brain, blood vascular, and spin problems (central nervous system). Additionally, it aids in the imaging of the liver, pelvis, heart, brain, and spine, as well as the musculoskeletal system.	69
**Gadoterate Dotarem, (Clariscan)**	Gadolinium contrast agent (paramagnetic)	Macrocyclic(ionic)	Brain and spine	This compound is authorized for body imaging as well as for the imaging of lesions in the brain and spine with aberrant blood-brain barrier or anomalous vascularity.	49
**Gadodiamide (Omniscan)**	Gadolinium contrast agent (paramagnetic)	Linear (non-ionic)	Brain, spine, chest, stomach, hip area, and other parts of the body	Gadodiamide can be helpful in a variety of MRI applications, such as hepatic, pelvic, cardiac, brain, spine, and musculoskeletal imaging, as an extracellular contrast agent.	70
** Gadobenate dimeglumine** **(MultiHance)**	Gadolinium-based Paramagnetic Contrast agent (Extracellular intravenous)	Linear(ionic)	Liver	Gadobenate dimeglumine is helpful as an extracellular contrast agent in a variety of MRI applications, such as imaging of the hepatic, pelvic, cardiac, brain, and spine, as well as the musculoskeletal system.	71
**Gadoteridol (ProHance)**	Gadolinium based contrast agent (paramagnetic)	Macrocyclic(non-ionic)	Brain and spine (CNS)	It has been approved by the USFDA and is used to see anomalies in the CNS and extramedullary tissues	72
**Gadoversetamide (OptiMARK)**	Gadolinium based contrast agent (Extracellular Paramagnetic)	Linear (non-ionic)	Brain, spine, and liver	Brain, spinal cord, and liver MRIs benefit greatly from its usage as a contrast agent.	73
**Gadofosveset trisodium** **(Ablavar or Vasovist)**	Gadolinium based contrast agent (Intravenous blood pool)	Linear(ionic)	Heart (angiography)	Gadofosveset serves as an injectable angiography imaging agent for the diagnostic imaging of cardiovascular system blood vessels. It has the potential to replace a number of the current invasive, catheter-based angiograms, x-ray tests, and thallium stress tests used to diagnose coronary artery disease. The substance may also be used to diagnose breast cancer, thrombosis, and peripheral vascular disease.	74
**Iohexol (Omnipaque)**	Iodinated Contrast agent	Non-ionic monomeric	Lumbar, thoracic, cervical, and total columnar region	It is utilized for computerized tomography contrast enhancement (myelography, cisternography, and ventriculography). There have been reports of its intravenous (IV), oral, and intrathecal usage based on the X-ray tomography applications where it was utilized to visualize the brain ventricles, urinary system, joints, arteries, and veins.	75
**Iopromide (Ultravist)**	Iodinated Contrast agent	Non-ionic monomeric	The brain, heart, head, blood vessels, stomach, and other parts of the body	Assessment of neoplastic and non-neoplastic lesions utilising CT of the head and body areas. Its principal uses include neoplasm viewing, peripheral coronary arteriography, and the cerebral areas of the brain.	75
**Iodixanol** **(Visipaque)**	Iodinated contrast agent	Non-ionic dimeric hydrophilic	Brain, heart, blood vessels, kidneys, bladder, and other parts of the body.	Iodixanol injection is used to help identify or diagnose issues with the body's blood arteries, kidneys, bladder, brain, heart, and other organs. It is frequently employed as a contrast material during coronary angiography, especially in patients with renal impairment because it is thought to be less harmful to the kidneys than the majority of other intravascular contrast materials. It is mostly utilized in CT applications for the investigation of cardiovascular disorders and the visualization of arteritis.	76
**Ioxaglate meglumine (Hexabrix)**	Iodinated contrast agent	Ionoic dimeric	Uterus, brain, heart, joints, urinary system, GI tract, Biliary and pancreatic ducts.	Imaging of the gastrointestinal tract, hysterosalpingography (which involves visualisation of the uterus and Fallopian tubes), angiography (which involves visualisation of blood vessels, including those of the brain and heart), arthrography (which involves visualisation of joints), urography (which involves visualisation of the urinary system), and endoscopic retrograde cholangiopancreatography (ERCP; which involves visualisation of the biliary and pancreatic ducts) are all procedures that make use of this technique.	77
**Iothalamate (CystoConray II)**	Iodinated contrast agent	Ionic monomeric	Brain, heart, blood vessels, joints, stomach, pancreas, bladder and head.	The injection of iothalamate meglumine is used to help identify or find issues with the bladder, pancreas, back, heart, head, blood vessels, stomach, joints, and other regions of the body.	78
**Iopamidol** **(Isovue)**	Iodinated contrast agent	Non-ionic monomeric	Heart	It was primarily employed in cardiovascular examinations, including cerebral, visceral, and coronary arteriography, as well as paediatric contrast enhancement of computer tomography cases.	79

**Table 2:** Theranostic platform for simultaneous imaging and therapy

Nanomaterials	Therapeutic moiety	Particle size	Disease state	Type of Imaging	Contrast agent	Concluding remarks	Ref.
Polymeric nanoparticles	-	5-10 nm	Colon cancer	Micro-CT and Fluorescence Molecular Tomography	Dy750	The non-invasive evaluation of nanomedicine biodistribution is much easier when anatomical μCT and molecular FMT are combined.	146
Iron oxide nanoparticles	Rutin	13 nm	Alzheimer's disease	MRI	Congo red	One promising new direction for AD theranostics is the use of Congo red/Rutin-iron oxide nanotheranostics.	147
Iron oxide nanoparticles	Curcumin	160-180 nm	Alzheimer's disease	MRI	-	An effective technique for the diagnosis and therapy of AD may be offered by this unique multifunctional nanomaterial.	148
Iron oxide nanoparticles	Ferumoxytol	-	Brain disease	MRI	Gadolinium	Nanoparticles of iron oxide as a potential substitute for MRI using gadolinium.	149
Liposomes nanoparticles	Doxorubicin	50 nm	Cancer	CT	Doxorubicin	This method and built system have the potential to pave the way for novel approaches to cancer treatment and targeted imaging.	150
Hyaluronic acid-functionalized iron (II) tungstate nanoparticles	-	91 nm	Cancer	MRI and CT	Metal tungstates	The biomedical applications of the synthesised nanoparticles, such as image-guided cancer theranostics and multimodal imaging, are practically limitless.	151
Polymeric nanoparticles	Paclitaxel	39-102 nm	Breast cancer	CT	Cy7.5	Metastatic breast cancer patients may be able to undergo CT imaging-guided treatment with this well-thought-out concept, which combines real-time CT imaging with safe and efficient anticancer medication.	152
Lanthanide-based NIR-IIb nanoparticles	-	38 nm	Brain cancer	MRI	Lanthanide	Nanoparticles can be more easily accumulated in orthotopic brain tumours using bioorthogonal labelling. These results have the potential to offer a simple, reliable method to aid in the early diagnosis of cancer.	153

## Abbreviations

MRI: Magnetic resonance imaging
CT: Computed tomography
PET: Positron emission tomography
CNS: Central nervous system
CAD: Coronary artery disease
PMRI: Preclinical magnetic resonance imaging
ICM: Iodinated contrast medium
MCT: Micro-computed tomography
MRICAs: Magnetic resonance imaging contrast agents
ECF: Extracellular fluid
DTPA: Diethylene tri-amine-penta-acetic acid
BPA: Blood pool agent's
SPIONs: Superparamagnetic iron oxide nanomaterial
Ln2O3: Lanthanide oxide
FL: Fluorescence
PDA: Polydopamine
FA: folic acid
HAase: Hyaluronidase
CE: Contrast-enhanced
DL: Deep learning
